# Immunological and pathobiological characteristics of a novel live *Salmonella* Typhimurium-vectored *Campylobacter* vaccine candidate for layer chickens

**DOI:** 10.3389/fvets.2025.1518231

**Published:** 2025-03-21

**Authors:** Jamieson B. Mcdonald, Emily Gan, Joel Cain, Sapna G. Thoduka, Joseph Lee, Ben Wade, Marta Mauri, Jon Cuccui, Brendan W. Wren, Nicolle H. Packer, Sarah L. Londrigan, Svenja Fritzlar, Sameera Mohotti, Gregory J. Underwood, Daniel M. Andrews, Thi Thu Hao Van, Robert J. Moore

**Affiliations:** ^1^School of Science, RMIT University, Bundoora, VIC, Australia; ^2^Bioproperties Pty Ltd, RMIT University, Bundoora, VIC, Australia; ^3^School of Natural Sciences, Macquarie University, Sydney, NSW, Australia; ^4^ARC Centre of Excellence in Synthetic Biology, Macquarie University, Sydney, NSW, Australia; ^5^Faculty of Infectious and Tropical Diseases, London School of Hygiene and Tropical Medicine, London, United Kingdom; ^6^Department of Microbiology and Immunology, The University of Melbourne at the Peter Doherty Institute for Infection and Immunity, Melbourne, VIC, Australia

**Keywords:** glycoconjugate, vaccine, *Salmonella* Typhimurium, *Campylobacter hepaticus*, spotty liver disease, chicken, live vaccine

## Abstract

**Introduction:**

Spotty liver disease (SLD) poses a significant economic and animal welfare threat to the global cage-free egg industry, primarily due to infection by the emerging pathogen *Campylobacter hepaticus*. SLD can lead to a significant decline in egg production and increased mortality rates. Antibiotics remain the most effective measure for controlling the disease. However, the rise of antibiotic resistance is a growing global concern for public health, promoting efforts to reduce antibiotic usage in animal production. Poultry vaccination offers an alternative approach to decreasing *C. hepaticus* levels. Although autogenous vaccines are in use in some countries with limited efficacy, no vaccine is currently licensed for widespread use.

**Methods:**

This study developed and characterized a live *Salmonella* Typhimurium vector strain designed to deliver the conserved *Campylobacter N*-glycan heptasaccharide as a target antigen against *C. hepaticus*.

**Results:**

The replacement of the *S.* Typhimurium *aroA* gene with the *Campylobacter pgl* locus attenuated the vaccine strain, allowing the conjugation of the heptasaccharide to *S.* Typhimurium endogenous lipopolysaccharide (LPS). Commercial layer hens vaccinated with the *S.* Typhimurium strain producing the *Campylobacter* heptasaccharide induced significantly higher IgY antibody titres specific to the *Campylobacter* heptasaccharide compared to the birds vaccinated with the vector strain not expressing the heptasaccharide. Modification of the *S.* Typhimurium endogenous LPS with the heptasaccharide had no significant impact on IgY antibody responses against *S.* Typhimurium.

**Discussion:**

This study provides evidence that using *S.* Typhimurium to deliver *Campylobacter* heptasaccharide is a feasible approach to providing bi-valent immunogenicity against both *S.* Typhimurium and *C. hepaticus*.

## 1 Introduction

*Campylobacter hepaticus* and *Salmonella enterica* subspecies serovar Typhimurium are significant Gram-negative pathogens affecting layer hens and humans, respectively. Eggs can be contaminated by *S.* Typhimurium through horizontal transfer and is a common cause of egg- and egg-product-related, self-limiting foodborne illness in humans (1, 2). In contrast, no human diseases have been linked to *C. hepaticus*. However, this bacterium causes Spotty Liver Disease (SLD) in layer chickens. Another *Campylobacter* species, *Campylobacter bilis*, found in only a small minority of field cases in Australia, was also confirmed to be a cause of SLD (3). This disease clinically presents as lesions that appear as numerous grey/white spots on the liver, elevated mortalities (up to 15%) (4), and a decrease in egg production (up to 35%) in flocks (5–7). The infection of layer chickens with these bacteria is a significant economic and animal welfare burden. The disease is most commonly seen in layer flocks raised in cage-free production environments, and it has been hypothesized that such birds are more susceptible to the faecal-oral route of infection (8). Cage-free egg production will continue to increase to meet the population demand and consumer preference for eggs produced from caged poultry (9, 10). Thus, controlling these pathogens on farms is crucial to maintaining healthy flocks, egg production levels, the safe supply of eggs to consumers, reducing foodborne infections, and mitigating the poultry industry’s economic problems.

Although cage-free farms employ biosecurity measures, feed additives, and environmental management to mitigate SLD risks, success remains limited. Currently, the most effective way of treating SLD-affected hens is to use antibiotics such as chlortetracycline, lincospectin, and amoxicillin. However, the rising threat of antibiotic resistance underscores a significant public health concern. Hence, the animal production industries are actively seeking alternative control methods, such as vaccination, to reduce antibiotic use without compromising animal health and productivity (11). Both live and killed poultry vaccines against *S.* Typhimurium infections are commercially available (12). Vaccination with live attenuated *S.* Typhimurium is favored over inactivated vaccines as it mimics the natural infection of the pathogen and possesses a repertoire of antigens capable of inducing cell-mediated, humoral, and mucosal immune responses (12, 13). Several live attenuated *S.* Typhimurium vaccines are available worldwide, effectively decreasing the risk poultry products pose to consumer infection. For example, Vaxsafe^®^ ST (STM-1) is a live attenuated *S.* Typhimurium vaccine produced by Bioproperties Pty Ltd. and registered for use in Australia. The vaccine strain, *S.* Typhimurium strain 82/6915, isolated from a chicken, has been attenuated via deletion of part of the *aroA* gene, which encodes an enzyme within the metabolic pathway responsible for synthesising essential aromatic amino acids (14). This vaccine strain also represents an ideal vector delivery system for other pathogen antigens, as its safety and efficacy have been thoroughly tested and evaluated (15–18).

Attempts have been made to develop a vaccine against *C. hepaticus* to reduce the incidence of SLD, but they have been largely ineffective. A killed *C. hepaticus* whole-cell vaccine has been tested in an experimental infection model; it induced serological responses, but titres were not maintained before clinical signs of SLD developed and, therefore, provided limited protection against disease (19). Although autogenous vaccines are employed in Australia and the US, their performance in commercial flocks remains undocumented in peer-reviewed literature. An alternative vaccination approach is to exploit live attenuated *Salmonella* as a vector to deliver *C. hepaticus* antigens to the chicken host’s immune system. Immunogenicity, conservation, and presentation are crucial considerations for choosing and evaluating antigens as vaccine targets. Traditionally, highly immunogenic and conserved protein antigens and their epitopes have been used as target molecules for vaccination against poultry pathogens. Other molecules, including carbohydrates, make up highly immunogenic structures that have been successfully used globally in the development of human glycoconjugate vaccines (20, 21) but are yet to be used in the poultry industry.

The *Campylobacter N*-glycan heptasaccharide is a promising vaccine antigen for multiple reasons. The carbohydrate is produced by the *N-*glycosylation system, which is encoded by the *pgl* locus and involves the action of several enzymes responsible for the construction and coupling of the *N-*glycan to acceptor proteins that possess the appropriate glycosylation sequon (D/E-X-N-X-S/T) (22–24). The *N-*glycan heptasaccharide post-translationally modifies multiple membrane proteins, acts as a key virulence factor, is immunogenic in humans and chickens, and is a conserved structure in *C. hepaticus, Campylobacter jejuni,* and *Campylobacter coli* species (25–32). The use of the *Campylobacter* heptasaccharide as a vaccine antigen has been achieved via the functional transfer of the *pgl* locus into a bacterial vector such as *Escherichia coli* (33) or *S.* Typhimurium (34). This method is termed protein glycan coupling technology, which can be used to produce recombinant glycoconjugate proteins and other heptasaccharide moieties (35–37).

The *N-*glycosylation pathway shares features with the O-antigen biosynthesis pathway used to construct rough lipopolysaccharide (LPS) in Gram-negative bacteria such as *E. coli* and *S.* Typhimurium. Crosstalk exists between these biochemical pathways when the *pgl* machinery is transferred and expressed in these bacteria (38–40). The lipid-linked carbohydrate structures generated by the *N-*glycosylation pathway can serve as building blocks for enzymes, including O-antigen ligase, WaaL, recruited for the transfer of O-antigen constituents from the undecaprenol lipid carrier to the core oligosaccharide of LPS (41, 42). This crosstalk permits conjugation of the *Campylobacter* heptasaccharide to *E. coli* and *S.* Typhimurium lipid A core oligosaccharide through a non-specific lipid carrier-associated mechanism. This transfer can be inhibited by inactivating the WaaL ligase (42), or alternatively, it can be leveraged to improve the surface display of the heptasaccharide antigen in a live bacterial vectored vaccine (34, 43). The efficacy of these constructs against *C. jejuni* infection in broilers has been explored, with varying levels of protection demonstrated. In one instance, IgY antibodies raised against *S.* Typhimurium displaying the *Campylobacter* heptasaccharide in birds vaccinated against *C. jejuni* could not recognize the *C. jejuni* heptasaccharide, and vaccination provided little protection against *C. jejuni* colonisation (34). Other studies have demonstrated specific IgY responses to the heptasaccharide when presented at the cell surface of a live attenuated carrier such as *E. coli* (43) and produced an immune response that significantly reduced *C. jejuni* colonisation in the gut of broiler chickens. *C. hepaticus* was recently shown to possess an *N-*linked protein glycosylation system responsible for the production of a heptasaccharide glycan likely to be identical at the structural level to that produced by *C. jejuni* (25), providing a rationale for the development of a vaccine that utilises the *N-*glycan heptasaccharide as a target to protect chickens from SLD. Inducing a systemic immune response through an appropriate vaccination strategy offers a promising approach to controlling *C. hepaticus* in poultry. This is primarily because the trafficking of *C. hepaticus* from the gastrointestinal tract to the gallbladder and liver is a likely mechanism of disease pathogenesis, potentially making it more vulnerable to the immune system of layer hens compared to *C. jejuni*, which typically resides in the gut lumen without causing overt disease.

This study aimed to characterize, validate, and immunologically assess a live *S.* Typhimurium vaccine candidate engineered to express the *Campylobacter* heptasaccharide at the core oligosaccharide as a part of *S.* Typhimurium 82/6915 endogenous LPS. The novel vaccine candidate strain was termed *S.* Typhimurium 82/6915*ΔaroA::pgl*.

## 2 Materials and methods

### 2.1 Vaccine strains, plasmids, and growth conditions

*Salmonella* Typhimurium 82/6915, STM-1, and *S.* Typhimurium 82/6915*ΔaroA::pgl* were grown at 37°C in Luria-Bertani (LB) or 2YT-M9 tryptone 14 g/L, yeast extract 10 g/L, MgSO_4_ 2 mM, CaCl_2_ 0.1 mM, and 1x M9 salts (Na_2_HPO_4_*·*7H_2_O 6.8 g/L, KH_2_PO_4_ 3 g/L, NH_4_Cl 1 g/L, NaCl 0.5 g/L). The media were supplemented as needed with ampicillin (Amp) or chloramphenicol (Cm) at a final concentration of 100 μg/mL and 30 μg/mL, respectively. The vaccine candidate was engineered following the methods described by Mauri et al. (35). Briefly, a suicide vector carrying the *C. jejuni* 81116 *pgl* locus terminally marked with a chloramphenicol resistance cassette surrounded by flippase recognition target (FRT) sites (for antibiotic resistance removal) and flanked by two 1 kb homology arms for integration into the *aroA* gene of the *S.* Typhimurium 82/6915 recipient strain was assembled. Sanger sequencing was performed to confirm the correct assembly of the suicide vector. The suicide vector was delivered via conjugation from *E. coli* MFDpir (Mu Free Donor, a diaminopimelic acid (DAP) auxotrophic strain) (44) into *S.* Typhimurium 82/6915, generating *S.* Typhimurium 82/6915*ΔaroA::pgl* integrant by allelic exchange. Antibiotic selection, polymerase chain reaction (PCR), and Illumina paired-end sequencing were used to confirm the integration of the *pgl* locus at the correct site in transformants. *Campylobacter* strains were grown on Brucella 5% horse blood agar (HBA) plates under microaerophilic conditions (5% O_2_, 5% CO_2_, and 90% N_2_) at 37°C for 48–72 h or in Brucella broth supplemented with L-cysteine (0.4 mM), L-glutamine (4 mM), and sodium pyruvate (10 mM) as described by Phung et al. (45).

### 2.2 Visualisation and structural characterisation of the vaccine glycoconjugate

#### 2.2.1 Initial lectin blotting and proteinase K digestion

Whole-cell lysates from bacterial strains were analyzed by sodium dodecyl sulphate-polyacrylamide gel electrophoresis (SDS-PAGE) and lectin blotting with soybean agglutinin (SBA). A total of 5 mL overnight cultures of STM-1 and *S.* Typhimurium 82/6915*ΔaroA::pgl* were grown at 37°C / 200 rpm, and *C. hepaticus* HV10^T^ was grown for 48 h on HBA plates under microaerophilic conditions. A total of 1 mL of each *S.* Typhimurium strain was harvested, *C. hepaticus* HV10^T^ plates were flooded with 2 mL PBS, harvested by gently scraping, and pelleted at 5,000 x *g* for 5 min, and washed three times in 1 mL PBS. Cell pellets were lysed as McDonald et al. (46) described with the addition of boiling for 5 min at 100°C. Supernatants from whole cell lysates were collected, and protein concentrations were determined using a Qubit assay kit (Thermo Fisher Scientific). Approximately 25 μg of protein extracts were suspended in 4x Laemmli sample buffer (Bio-Rad) containing 50 mM dithiothreitol or the equivalent amount of whole cell lysates treated with proteinase K (100 μg/mL) at 55°C for 30 min. Samples were heated for 5 min at 95°C, loaded on 8–16% precast polyacrylamide gels (Bio-Rad), and separated at 200 V for 45 min. SDS-PAGE gels were either developed using SimplyBlue Safe stain (Invitrogen) or transferred to a polyvinylidene fluoride (PVDF) membrane using an iBlot™ Dry Blotting system (Invitrogen). Lectin blotting was performed as previously described by McDonald et al. (25), using SBA as the primary detection reagent and streptavidin-HRP as the secondary detection solution. Signals were detected using tetramethylbenzidine (TMB), and blots were imaged on a ChemiDoc imaging system (Bio-Rad).

#### 2.2.2 Purification and visualization of *Salmonella* Typhimurium 82/6915Δ*aroA::pgl* LPS

To determine whether the *Campylobacter* heptasaccharide had integrated as a part of *S.* Typhimurium 82/6915*ΔaroA::pgl* endogenous LPS, the LPS from this strain was isolated and analyzed. LPS was extracted from 3 L of freshly grown overnight cultures of *S.* Typhimurium 82/6915*ΔaroA::pgl* and a 1 L overnight culture of *S.* Typhimurium STM-1 using a method adapted from (47, 48). Briefly, 3 × 1.0–1.5 g cell dry weight of *S.* Typhimurium 82/6915*ΔaroA::pgl* and 1.0 g cell dry weight of STM-1 were washed with 20 mL PBS containing 0.15 mM CaCl_2_ and 0.5 mM MgCl_2_. Cell suspensions were pelleted and resuspended in 20 mL of the same buffer. Cell suspensions were boiled at 100°C for 20 min and mixed every 5 min by inverting tubes. Cell suspensions were cooled, treated with 200 μg/mL proteinase K, and heated at 59°C for 3 h. DNase and RNase were added to samples at a final concentration of 40 μg/mL and 80 μg/mL, respectively, in the presence of 20 μL of 20% MgSO_4_ and 80 μL of chloroform, and the mixture was heated at 37°C for 1 h. Phenol/TRIS solution (20 mL) was heated to 65°C, added to enzymatically treated cell lysates (20 mL), and incubated at 65°C for 15 min with occasional vortexing and shaking. Cell suspensions were cooled on ice and centrifuged at 8500 x *g* for 15 min. Supernatants were transferred to fresh tubes, and the phenol phase was re-extracted by adding 20 mL of Milli-Q water. Sodium acetate at a final concentration of 500 mM and 10 volumes of 95% ethanol were added to LPS extracts and stored at −20°C overnight to precipitate LPS. Samples were centrifuged at 2,000 x *g*, 4°C for 10 min; supernatants were removed, and pellets were resuspended in 3 mL of Milli-Q water. Samples were heated at 75°C with vortexing until LPS samples were solubilized. A total of 2 mL of the LPS solutions were dialysed against Milli-Q water with three changes at 1 h intervals to remove residual phenol and other contaminants. A total of 2 mL of the LPS extracts were lyophilised and stored at 4°C.

Lyophilised LPS samples were resuspended in 1 mL of Milli-Q water, and 5 μL of each sample was added to 5 μL of 4x Laemmli sample buffer and 10 μL of PBS and loaded onto 8–16% precast polyacrylamide gels and separated at 100 V for 80 min. LPS extracts were visualized by direct staining either with the Pierce Silver stain kit (Thermo) or Pro-Q Emerald 300 Lipopolysaccharide Gel Stain Kit (Thermo) following the manufacturer’s instructions. Lectin blotting of the LPS samples was also performed to determine whether LPS was modified with the *Campylobacter* heptasaccharide.

#### 2.2.3 Extraction of *Salmonella* Typhimurium 82/6915Δ*aroA::pgl* and STM-1 LPS (small scale) for immunoblotting and mild acid hydrolysis

Smaller amounts of LPS were extracted from *S.* Typhimurium 82/6915*ΔaroA::pgl* and STM-1 as described by Davis and Goldberg (49). Briefly, 1.5 mL suspensions of bacterial strains at an OD_600_ = 0.5 were pelleted at 10,600 x *g* for 10 min, the supernatant removed, and the pellets resuspended in 200 μL of 1x Laemmli sample buffer containing 175 mM dithiothreitol (DTT). Cell pellets were lysed by heating at 100°C for 15 min. Lysed pellets were treated with 500 μg/mL proteinase K at 59°C for 3 h, and 200 μL of Tris-saturated phenol was added to each sample, vortexed, and heated at 65°C for 15 min. Samples were cooled, and 1 mL of diethyl ether was added. Samples were vortexed and centrifuged at 20,600 x *g* for 10 min. The bottom layer containing LPS was collected and subjected to a second round of Tris-saturated phenol and diethyl ether treatment and stored at −20°C until analysis.

Mild acid hydrolysis of LPS was performed to cleave the Kdo bond between the lipid A and the core oligosaccharide region of LPS as described by Batley et al. (50) with some modifications. Briefly, 40 μL of extracted LPS samples were treated with 1 or 2% glacial acetic acid (80% w/v) and heated at 100°C for 2 h. Samples were neutralized by the addition of 1 or 2% 10 M NaOH, respectively. A total of 10 μL of each LPS sample was separated by SDS-PAGE and visualized by direct staining either with the Pierce Silver Stain Kit (Thermo) or the Pro-Q Emerald 300 Lipopolysaccharide Gel Stain Kit (Thermo). Lectin blotting of LPS samples was also performed, as McDonald et al. (25) described, to determine whether LPS was modified with the *Campylobacter* heptasaccharide.

#### 2.2.4 LPS glycomics

*S.* Typhimurium 82/6915*ΔaroA::pgl* and STM-1 lyophilised LPS samples were reconstituted in ultrapure water to 10 mg/mL. In some experiments, low molecular weight LPS was further purified by passing hot phenol extracts through a 10 kDa molecular weight cut-off spin filter (Merck Millipore) according to the manufacturer’s instructions. Reconstituted LPS extracts were acidified with the addition of acetic acid to a concentration of 1% (v/v), after which samples were subjected to partial hydrolysis by heating at 95°C for 90 min. Samples were lyophilised in a SpeedVac (Savant), reduced by reconstitution in 0.5 M NaBH_4_ and 50 mM KOH, and incubated overnight at 50°C.

Following reduction, samples were diluted to a volume of 100 μL with ultrapure water, acidified with 1% acetic acid, and then desalted using HyperCarb™ porous graphitic carbon stage-tips (Thermo Scientific). Following the equilibration of columns with 0.1% trifluoroacetic acid (TFA), samples were loaded onto the stage tips three times. The columns were then washed with three volumes of 0.1% TFA. Subsequently, glycans were eluted using a solution of 50% acetonitrile (MeCN) and 0.1% TFA and were lyophilised prior to analysis.

#### 2.2.5 Liquid chromatography–tandem mass spectrometry

For the analysis of free/released glycans, samples were reconstituted in ultrapure water and loaded onto a 3 cm x 1 mm inner diameter (i.d.) x 3 μm Hypercarb™ porous graphitic carbon (PGC) column (Thermo Scientific) in 100% buffer A (10 mM NH_4_HCO_3_) at 20 μL/min using an Agilent 1290 Infinity LC system. Glycans were eluted by altering the mobile phase to 14% buffer B (70% MeCN, 10 mM NH_4_HCO_3_) over a 1 min linear gradient, after which the mobile phase was altered to 40% over a 36 min linear gradient directly into a linear triple quadrupole Velos™ Pro mass spectrometer (Thermo Scientific). The instrument was configured to perform one full scan MS experiment (scan range 570–2000 *m/z*, automatic gain control [AGC] of 3e4, and maximum IT of 100 msec) with the top 5 precursors in fulfilment of the selection criteria (dynamic exclusion window of 15 s) selected for MS/MS (scan range 150–2000 *m/z*, AGC of 1e4, maximum IT of 100 msec, isolation window 1.4 *m/z*, normalized collision energy [NCE] set as 33). Ions pertaining to glycans and/or glycoconjugates were identified by the presence of relevant oxonium ions (e.g., 204 *m/z*), after which glycan structural configuration was confirmed manually.

#### 2.2.6 Fluorescence microscopy and flow cytometry

Overnight cultures of STM-1 and *S*. Typhimurium 82/6915*ΔaroA::pgl* were grown at 37°C with shaking at 200 rpm, and *C. hepaticus* HV10^T^ was grown for 48 h on HBA plates under microaerophilic conditions. A total of 1 mL suspensions of bacterial strains at OD_600_ = 1.0 were pelleted at 8,000 x *g* for 10 min and washed twice with 1 mL PBS. Bacterial pellets were resuspended, fixed in 1 mL of 3% paraformaldehyde, and incubated at RT for 30 min. Fixed cells were pelleted and washed three times with 1 mL PBS. Cell pellets were resuspended in PBS to a final OD_600_ of 0.2–0.3 and stored at 4°C. Fixed cells were pelleted at 8,000 x *g* for 10 min, and the supernatants were discarded. Cells were blocked in 5% BSA for 1 h at RT or overnight at 4°C. The blocking solution was discarded, and cells were incubated for 1 h in the dark with either 25 μg/mL SBA fluorescein (Vector Laboratories) or 25 μg/mL FITC anti-*Salmonella* antibody (Abcam) diluted in PBST (PBS plus 0.1% Tween 20) containing 1% BSA. The unbound antibody was removed by washing the cells three times in 1 mL PBS.

For flow cytometry, cells were resuspended in PBS to approximately 10^6^ cfu/mL, and a total of 10,000 events were acquired and analyzed for each sample using a Beckman Cytoflex and CytExpert 2.5 software. For fluorescence microscopy, samples were counterstained with DAPI at a final concentration of 10 μg/mL in PBS-1% BSA solution for 15 min in the dark at RT. Cells were pelleted and washed twice in 1 mL PBS. Finally, cells were resuspended in 50 μL PBS and diluted 1/100. A total of 10 μL of stained bacteria were added onto coverslips and allowed to air dry in the dark for 30 min. Coverslips were then mounted onto Superfrost microscopic slides with 15 μL of Mowiol DABCO mounting solution and incubated at RT in the dark for 30 min before examination using a Leica DM2500 epifluorescence microscope. Images were captured using LAS software (V4.1; Leica Microsystems).

### 2.3 Assessment of the immunological characteristics of the *Salmonella* Typhimurium 82/6915*ΔaroA::pgl* vaccine in layer hens

#### 2.3.1 Animal experiments and study design

An animal study followed the protocol approved by the RMIT University Biosafety Committee (NLRD 2002) and the CSIRO Animal Ethics Committee, AEC approval 2050. A total of 45 six-week-old Hy-Line Brown commercial layer hens from a *Campylobacter* and *Salmonella-*free flock were obtained from a commercial layer pullet farm. Animal experiments were conducted at the Werribee Animal Facility of the Australian Animal Health Laboratory (CSIRO). Birds were randomly allocated into three groups and reared on deep litter (softwood shavings) in floor pens, identified using wing tags, and acclimatized for one week before conducting the trial. Cloacal swabs were collected before arriving at the facility. DNA was extracted from the swabs using a Maxwell RSC Buccal swab kit (Promega) according to the manufacturer’s instructions to confirm the absence of *C. jejuni* by PCR as described by Eyers et al. (51), which was also used to confirm the absence of *C. hepaticus*. The absence of *S.* Typhimurium was confirmed by PCR, as described below. The study involved the use of the groups detailed in Table 1.

**Table 1:** Treatment groups.

Bird Strain	Vaccine group	No. of birds	Purpose
Hy-Line Brown layers (7 weeks)	Group 1: Unvaccinated	15	Negative controls
	Group 2: STM-1 */* Nobilis™ EDS-ND^a^	15	Vaccine carrier controls
	Group 3: *Salmonella* Typhimurium 82/6915*ΔaroA::pgl /* Nobilis™ EDS-ND^a^	15	Experimental group

^a^Nobilis™ EDS-ND is a commercial, multivalent egg drop syndrome/Newcastle disease killed vaccine (Nobilis® EDS + ND, MSD Animal health) co-administered to mimic in-field practices.

**Figure 1:**
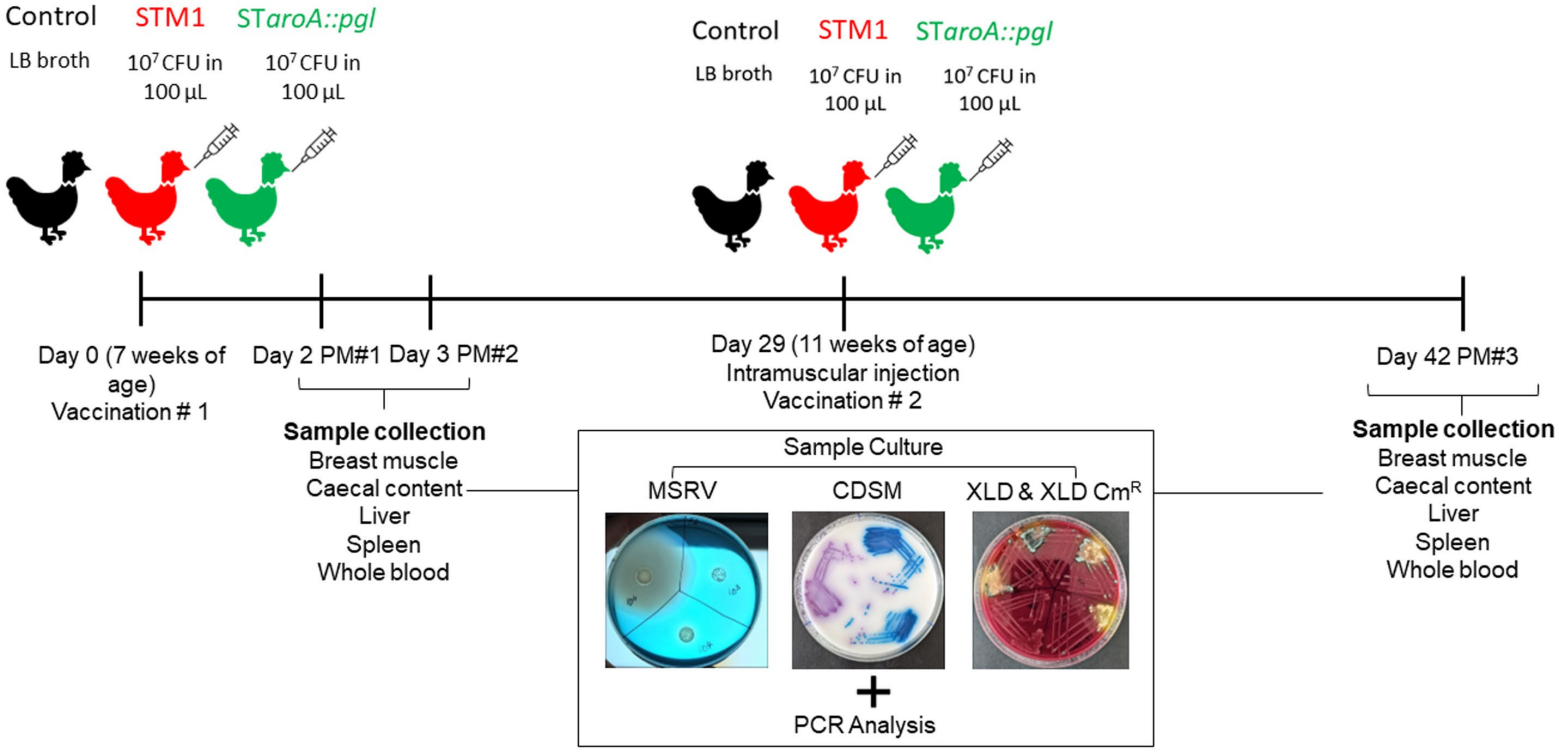
A flowchart of experimental design and sample collection points. Whole blood samples were taken on postmortem (PM) days and at weekly intervals throughout the trial.

To evaluate vaccine strain colonisation and persistence at specific sites, host tissues and caecal content samples were processed and enriched in tryptone soy broth. These samples were then plated on modified semi-solid Rappaport-Vassiliadis agar (MSRV), Oxoid *Salmonella* Chromogenic medium (OSCM), and Xylose Deoxycholate (XLD) agar, both with and without chloramphenicol. This approach facilitated the selection of *S.* Typhimurium 82/6915Δ*aroA::pgl* (Cm^R^) from STM-1, which does not carry Cm^R^. Whole blood samples were taken weekly from the same five birds from each group for serological analysis. The experimental plan and study design are depicted in Figure 1.

#### 2.3.2 Vaccination with *Salmonella* Typhimurium 82/6915Δ*aroA::pgl* and STM-1

To prepare the vaccine strains, *S.* Typhimurium 82/6915Δ*aroA::pgl* was revived from −80°C glycerol stocks, streaked onto LB agar, and grown overnight (16–18 h) at 37°C. A single colony of *S.* Typhimurium 82/6915Δ*aroA::pgl* was subcultured into 5 mL LB broth and also grown overnight (16–18 h) at 37°C with shaking at 200 rpm. The optical density at 600 nm (OD_600_) of the *S.* Typhimurium culture was measured, and the culture was diluted/adjusted to approximately 1 × 10^7^ CFU/100 μL, assuming an OD_600_ of 1 corresponds to approximately 1 × 10^9^ CFU/mL. For STM-1 preparation, vaccine vials were reconstituted in distilled water and diluted/adjusted to the same concentration. Cell counts were confirmed by serial dilution and overnight incubation on *Salmonella* XLD selective agar plates (Thermo Scientific). Chickens in groups 2 and 3 were vaccinated via intramuscular (IM) injection with 100 μL of cultures containing 10^7^ CFU. This route of administration was chosen because IM injection of STM-1 had previously been shown to significantly improve antibody responses to vaccination (18) and is routinely used for administering multiple vaccines to pullets between 8 and 14 weeks of age. Vaccines were co-administered with 500 μL of a commercial multivalent egg drop syndrome/Newcastle disease killed vaccine (Nobilis® EDS + ND, MSD Animal Health) to simulate in-field conditions. Co-administration involved first drawing 500 μL of Nobilis® EDS + ND into the syringe, followed by 100 μL of *S.* Typhimurium vaccines, and injecting the unmixed preparation into the breast muscle. Mock/unvaccinated birds received 100 μL of LB broth. Birds were vaccinated twice, at 7 and 11 weeks of age.

Cloacal swabs and whole blood from wing veins were collected from five birds per treatment group on days 7, 14, 21, 28, and 35 of the trial to assess *Salmonella* shedding and monitor the development of antibody responses to vaccination by ELISA. On study days 2, 3, and 42, five birds from each group were euthanised by CO_2_ asphyxiation in accordance with approved procedures before whole blood collection by cardiac puncture and post-mortem examination. Whole blood samples were centrifuged at 1000 x *g* for 10 min, and the collected serum was stored at −20°C for subsequent IgY analysis by immunoblotting and ELISA. Breast muscle tissue at the injection site, caecal content, cloacal swabs, and liver and spleen samples were harvested to determine the localisation and persistence of the vaccine strains post-intramuscular injection. Localisation and persistence were assessed by culturing tissue samples and extracting DNA for PCR analysis.

#### 2.3.3 PCR analysis of vaccine localization and persistence

##### 2.3.3.1 *aroA*-*pgl* PCR primer design and amplification

Primers were designed to specifically detect the *pgl* locus integration in the *S.* Typhimurium 82/6915*ΔaroA::pgl* vaccine strain (Table 2). PCR was carried out in a final volume of 25 μL using MyFi mix (Bioline), primers at a final concentration of 200 nM each, and 1 μL of template DNA. The PCR cycling conditions were as follows: 95°C for 3 min, 30 cycles at 95°C for 30s; annealing temperature of 65°C for 45 s and 72°C for 2 min, followed by a final extension of 72°C for 7 min to produce a 1.5 kB amplicon.

**Table 2:** Primers and probes used for qPCR assays.

Gene	Primer/probe	Sequence (5′ – 3′)
*pgl*	Oligo1-*aroA*/F *pgl* insert/R	GCCAGTTGATTCTCGCTG TCCAAAGTGCCGTGGTTTTG
*invA*	STM0159-F	ATGATGCCTTTTGCTGCTTT
	STM0159-R	TCCCAGCTCATCCAAAAA
	STM0159-P	(FAM)-CAGCCATCATCAGCGTAAGA-(BHQ-1)
*aroA* insertion	*aroA*/IS10-2F	GGTGTAATTGATCCCCAACG
	*aroA*/IS10-2R	ATTTTTGGCGAAACCATTTG
	*aroA/*IS10-P	(FAM)-CAGGAGCAAAGCACTGATGA-(BHQ-1)

##### 2.3.3.2 pan-ST real-time quantitative PCR (qPCR)

A TaqMan probe-based qPCR assay was employed to detect *S.* Typhimurium (including STM-1) and environmental isolates using the primer set STM0159-F and STM0159-R and the STM0159-P probe, which is labeled with a FAM fluorophore at one end and a BHQ-1 quencher at the other (see Table 2). The qPCR was performed on a Rotor-Gene Q 2Plex system (QIAGEN) under the following cycling conditions: initial denaturation at 95°C for 2 min, followed by 45 cycles of denaturation at 95°C for 5 s and annealing/extension at 60°C for 10s. The reaction mixture for each sample, with a total volume of 25 μL, included QuantiNova Probe master mix (QIAGEN), 200 nM of each primer and probe, and 5 μL of the diluted DNA template (1,5 dilution). DNA from *S.* Typhimurium 82/6915 DNA was included in each run as a positive control.

##### 2.3.3.3 STM-1 quantitative qPCR

An STM-1-specific TaqMan qPCR assay was used to detect the presence /absence of the STM-1 vaccine strain in samples. This assay employed the primer pair *aroA*/IS10-2F and *aroA*/IS10-2R, along with the *aroA*/IS10-P probe, which is labeled with a FAM fluorophore and a BHQ-1 quencher (see Table). Each qPCR reaction mix contained QuantiNova Probe master mix (QIAGEN), 200 nM of each primer, 100 nM of the probe, and 5 μL of diluted DNA template (1:5 dilution). Amplification was conducted on a Rotor-Gene Q 2Plex instrument under the following cycling conditions: initial denaturation at 95°C for 2 min, followed by 45 cycles of 95°C for 5 s, 62°C for 10 s, and 72°C for 5 s. DNA from STM-1 DNA was included in each run as a positive control.

#### 2.3.4 Processing of tissue and caecal content samples

Breast muscle at the injection site, liver, spleen, and caecal content samples were processed for *S.* Typhimurium 82/6915Δ*aroA::pgl* and STM-1 isolation by culturing as described in (18, 52, 53). Briefly, samples were pulverized using a scalpel and tweezers, and homogenized tissue samples were placed in 6-well culture plates containing 4 mL tryptone soy broth and incubated at 37°C for 18 h while gently shaking. Three 30 μL spots of enriched cultures were plated onto MSRV agar and grown at 37°C for 24 h. Cultures were subcultured onto OSCM and grown at 37°C for 18 h. Purple colonies were presumed to be *Salmonella,* and individual colonies were subcultured onto XLD and XLD Cm_30_ plates and grown at 37°C for 18 h. STM-1 can be differentiated from wild-type *Salmonella* due to its inability to produce H_2_S on XLD; thus, colonies appear white and lack the black centres characteristic of wild-type *Salmonella* grown on XLD (54). To differentiate STM-1 from *S.* Typhimurium 82/6915Δ*aroA::pgl,* colonies were selected on XLD Cm_30_ antibiotic plates. In addition, DNA was extracted from cloacal swabs and tissue samples using the Maxwell RSC Buccal Swab & PureFood GMO DNA kit (Promega), respectively, as per the manufacturer’s instructions. As mentioned above, DNA extracts were used as templates for PCRs designed to detect amplicons specific to each vaccine strain.

#### 2.3.5 Immunoblotting to assess IgY antibody responses specific to the *Campylobacter* heptasaccharide

Immunoblotting was performed on extracted LPS samples as previously prepared. Briefly, 2 μL of LPS extracts were loaded onto 8–16% precast polyacrylamide gels and subjected to SDS-PAGE at 100 V for 75 min. The LPS samples were then transferred to PVDF membranes, which were blocked with 5% BSA for 1 h at room temperature (RT) while rotating on a roller mixer. Blots were then probed with 1:500 of pooled sera from birds in group 1 (unvaccinated) and group 3 (vaccinated with *S.* Typhimurium 82/6915Δ*aroA::pgl*) taken at day 42. Sera were diluted in PBST (0.05% Tween20) containing 1% BSA, 100 μL of total STM-1 LPS extracts, and 100 μL of STM-1 OD600 = 1 WCL to pre-adsorb any antibodies with cross-reactive responses to *S.* Typhimurium LPS. The blots were incubated for 1 h at RT.

Following incubation, the blots were washed three times for 5 min each in PBST (0.05% Tween 20) and subsequently incubated with a 1:5000 dilution of rabbit anti-chicken IgY (H + L) secondary antibody conjugated to horseradish peroxidase (HRP), diluted in PBST (0.05% Tween 20) containing 1% BSA for 1 h at RT. The blots were again washed three times for 5 min each in PBST (0.05% Tween 20), followed by three 5-min washes in PBS and a final single 5-min wash in Milli-Q water. Detection was carried out using a chromogenic TMB substrate.

#### 2.3.6 IMAC purification of *N*-glycoprotein to use as a coating antigen for indirect ELISA

IgY antibody responses specific to the *Campylobacter* heptasaccharide region of the vaccine were quantified using indirect ELISA. The coating antigen used was a *Campylobacter N-*glycoprotein CjaA_ChuA (9), modified with nine *Campylobacter N*-glycans and a hexahistidine (His_6_) tag. CjaA_ChuA is a fusion protein that combines the antigenic *Campylobacter N-*glycoprotein, CjaA (55), with the predicted antigenic epitope of the *Campylobacter* ChuA protein, incorporating eight additional sequons for *N-*glycan attachment. This glycoprotein and its unglycosylated equivalent were purified from *S.* Typhimurium 82/6915Δ*aroA::pgl* [pUC57CjaA_ChuA (9)] (glycosylated) and *S.* Typhimurium 82/6915 [pUC57CjaA_ChuA (9)] (non-glycosylated), respectively, using immobilized metal affinity chromatography (IMAC).

Briefly, *S.* Typhimurium 82/6915Δ*aroA::pgl* [pUC57CjaA_ChuA (9)] and *S.* Typhimurium 82/6915 [pUC57CjaA_ChuA (9)] cultures were grown overnight in LB broth supplemented with ampicillin (100 mg/mL) at 37°C with shaking at 200 rpm. Pellets were harvested by centrifugation at 10,000 × *g* for 10 min. The pellets were then sonicated for 4 min (20 s on, 10 s off at 40% frequency) in Ni-NTA lysis buffer (300 mM NaCl, 50 mM NaH_2_PO_4_, 10 mM imidazole, pH 8). After sonication, the lysates were centrifuged at 7,000 × *g* for 10 min to collect the supernatant.

For His-tag purifications, 1–2 mL of Ni-NTA beads (Qiagen) were used for every 2–4 litres of culture media equivalent supernatant. The beads were placed in an appropriately sized gravity column washed with 10 column volumes (CV) of Ni-NTA wash buffer (300 mM NaCl, 50 mM NaH_2_PO_4_, 20 mM imidazole, and pH 8). The beads were then added to the supernatant and incubated with rolling for 1 h at RT or overnight at 4°C.

After incubation, the beads were loaded back into the column and washed with wash buffer. For glycosylated samples, the beads were washed with 3 CV of 20 mM imidazole wash buffer, while unglycosylated samples were washed with 2 CV of 20 mM imidazole wash buffer, followed by 2 CV of 30 mM imidazole wash buffer. Glycosylated samples were then eluted with a buffer containing 300 mM NaCl, 50 mM NaH_2_PO_4_, and 250 mM imidazole, pH 8. Unglycosylated samples were eluted with a buffer containing 300 mM NaCl, 50 mM NaH_2_PO_4_, and 80 mM imidazole, pH 8. The eluates were then desalted using Cytiva G-25 MidiTrap columns with PBS.

The glycosylated protein underwent an additional Ni-NTA purification step using Ni-NTA beads, repeating the previous steps with a 0.5–1.0 mL Ni-NTA bead bed. Finally, electroelution was used to remove any contaminating *S.* Typhimurium proteins from the purified protein samples. A total of 800 μL of desalted Ni-NTA purified unglycosylated sample was loaded into two pre-cast 12-lane gels and run at 200 V for 32 min. The first lane of each gel was cut and stained/destained with Coomassie, while the remaining lanes were left in SDS-Tris-glycine buffer. The stained lane was paired with the unstained lanes from the same gels, and the known 37 kDa band was excised from the gel. The unstained excised gel strip was inserted into a Slide-A-Lyzer™ G3 Dialysis Cassette, 20 K MWCO. Cassettes were placed into one side of a DNA gel electrophoresis tank in chilled SDS-Tris-glycine buffer and electroeluted at 250 V for 2 h. Electroelution was paused every 30 min to replace the buffer with fresh, chilled buffer to prevent overheating. Cassettes were then dialysed against PBS, and samples were extracted, 0.22 μm filtered, and concentrated using an Amicon® Ultra Centrifugal Filter, 10 kDa MWCO.

1 mL of SBA-bound agarose beads (Vector Laboratories, AL-1013-2) were washed with 10 CV of SBA Buffer (150 mM NaCl, 50 mM HEPES, pH 8) to obtain highly purified glycoprotein. The beads were loaded into the desalted, twice Ni-NTA purified glycosylated sample (1–1.5 mL of sample for every 1 L of culture starting material) and incubated with rolling for 1 h at RT or overnight at 4°C. The beads were loaded into a 2 mL gravity column and washed with 3 CVs of SBA Buffer. The sample was pre-eluted with 0.3 CV of Glycoprotein Eluting Solution (Vector Laboratories, ES-2100) before eluting with 3 CV of Glycoprotein Eluting Solution. The eluate was added to a Slide-A-Lyzer™ G3 Dialysis Cassette, 20 K MWCO, and dialysed against PBS. Samples were extracted from the dialysis cassette, 0.22 μm filtered, and concentrated using an Amicon® Ultra Centrifugal Filter, 10 kDa MWCO.

Protein concentrations were determined using a Qubit assay kit and separated by 8–16% SDS-PAGE. Protein expression and glycosylation status were verified by western blotting and SBA lectin blotting, respectively. For western blotting, gels were transferred to PVDF membranes and blocked in 5% BSA at RT for 1 h. Blots were then probed with mouse anti-his [1:3000] in PBST (0.5% Tween_20_) containing 1% BSA and incubated at RT for 2 h while rotating. Blots were washed three times in PBST (0.05% Tween_20_) followed by incubation in [1: 5000] goat anti-mouse IgG in PBST containing 1% BSA for 1 h at RT. Blots were then washed three times for 5 min in PBST and PBS and 5 min in Milli-Q water. Membranes were incubated in TMB for chromogenic detection of his-tagged proteins.

#### 2.3.7 Serological ELISAs

Serum IgY vaccine-specific responses were quantified using ELISA. To measure antibody (IgY) responses against *S.* Typhimurium, a *Salmonella* group B antibody test kit (BioChek) was used following the manufacturer’s instructions. A 96-well plate ELISA assay was developed to quantify serum IgY responses specific to the *Campylobacter* heptasaccharide in which serum was tested against unglycosylated or glycosylated forms of CjaA_ChuA (9) as the capture antigen. Briefly, 96-well plates were coated with 50 μL of purified CjaA_ChuA (9) or unglycosylated CjaA_ChuA (9) and resuspended in a carbonate–bicarbonate buffer at a final concentration of 1 μg/mL and incubated at RT for 2 h. Wells were blocked with 200 μL of 5% BSA for 2 h at RT. While blocking, sera samples were diluted 1/100 in 1% BSA and pre-adsorbed with 4.5 μg/mL *S.* Typhimurium 82/6915 WCL and 7 μg/mL of purified unglycosylated CjaA_ChuA (9) for 2 h at RT to reduce any potential cross-reactivity to *Salmonella* and CjaA_ChuA (9), respectively. Plates were washed three times with 200 μL of PBST (0.05% Tween 20), and 100 μL of 1:100 pre-adsorbed sera were added to each well in triplicate, and plates were incubated at RT for 1.5 h. Control wells were maintained for both capture antigens to which no serum was added. Plates were washed as above, and 100 μL rabbit anti-chicken IgY (H + L)– HRP [1:10000] was added to each well. Plates were incubated at RT for 1.5 h, washed as above, and 50 μL of TMB substrate was added to the plates. Plates were incubated at RT for 10 min in the dark, and the reaction was stopped with the addition of 50 μL of 1 M hydrochloric acid to each well. The optical density at 450 nm was measured using a plate reader with background corrections to absorbance values using the control wells to which each respective antigen was added, but sera were excluded.

#### 2.3.8 Statistical analyses

The distribution of data was evaluated using the Shapiro–Wilk normality tests. Statistical analyses of IgY titers and OD_450 nm_ readings between groups of *S.* Typhimurium 82/6915Δ*aroA::pgl,* STM-1, and mock-vaccinated chickens were performed in GraphPad Prism version 10.00 using a one-way ANOVA test with Dunnett’s multiple comparisons test. Data were represented as mean values with standard error of the mean. *p* values <0.05 were deemed to be statistically significant. Statistical analyses of IgY titres against *S.* Typhimurium between groups of *S.* Typhimurium 82/6915Δ*aroA::pgl-*vaccinated, STM-1-vaccinated, and mock-vaccinated chickens were performed in GraphPad Prism version 9.00 (GraphPad Software) using the Kruskal-Wallis test followed by Dunn’s multiple comparisons test. p values <0.05 were deemed to be statistically significant. Data were represented as median values with 95% confidence intervals.

## 3 Results

### 3.1 Functional validation of *Salmonella* Typhimurium expressing the *Campylobacter* heptasaccharide

#### 3.1.1 Initial screening of vaccine strain

**Figure 2:**
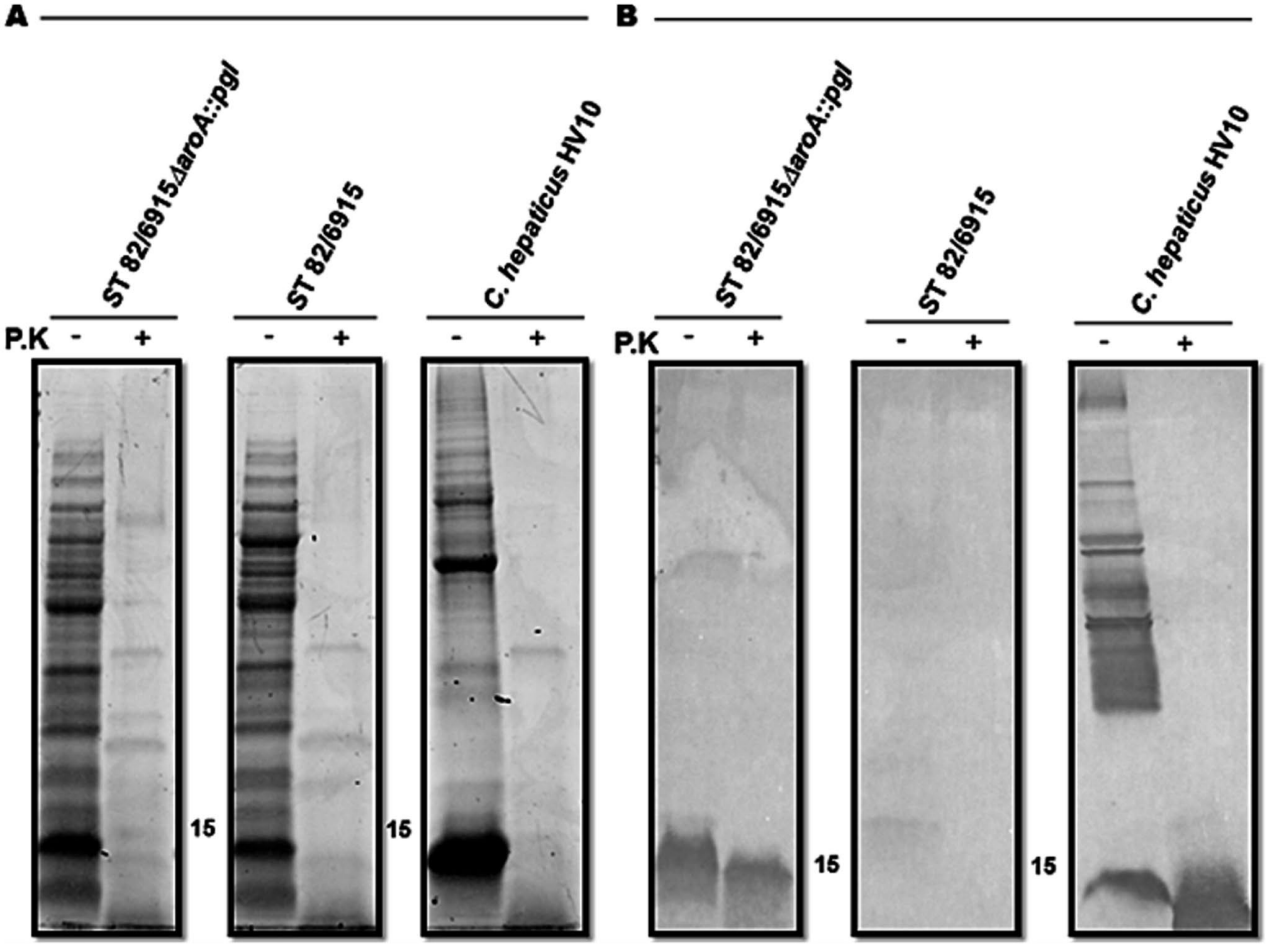
Screening of *Salmonella* Typhimurium 82/6915*ΔaroA::pgl* crude whole cell lysates. **(A)** SDS-PAGE of *Salmonella* Typhimurium 82/6915*ΔaroA::pgl*, *Salmonella* Typhimurium 82/6915 (negative control), and *C. hepaticus* HV10^T^ (positive control) whole cell lysates, which contained approximately 25 μg of protein treated with or without proteinase K (P.K) **(B)** SBA lectin blot binding profiles of *Salmonella* Typhimurium 82/6915*ΔaroA::pgl, Salmonella* Typhimurium 82/6915 (negative control), and *C. hepaticus* HV10^T^ (positive control) whole cell lysates contained approximately 25 μg of protein treated with or without proteinase K. The 15 displayed represents the size in kDa.

To demonstrate that the *Campylobacter* heptasaccharide was expressed in *S.* Typhimurium 82/6915*ΔaroA::pgl,* lectin blotting was performed using the SBA lectin that specifically binds to the *N-*acetylgalactosamine (Gal*N*Ac) residues of the heptasaccharide. A functional *pgl* operon may permit the conjugation of the heptasaccharide to either protein at an asparagine residue within naturally occurring *S.* Typhimurium *N-*glycosylation sequons or to the core oligosaccharide of *S.* Typhimurium LPS. Incorporation as a part of the LPS can occur due to the relaxed specificity of *S.* Typhimurium O-antigen ligase, WaaL, toward saccharide substrates and the addition of these sugars to the lipid A core of LPS. Thus, the binding of SBA to *S.* Typhimurium 82/6915*ΔaroA::pgl* whole cell lysates and the equivalent extracts treated with proteinase K were investigated (Figure 2).

Samples treated with proteinase K resulted in the digestion of proteins (Figure 2A) as expected. Lectin blotting of proteinase K-treated and untreated samples of *S.* Typhimurium 82/6915*ΔaroA::pgl* produced a dominant signal at 15 kDa (Figure 2B), which was absent in the proteinase K-treated whole cell lysate of *S.* Typhimurium lacking the *pgl* locus (*S.* Typhimurium 82/6915). This indicated that a *Campylobacter* saccharide containing Gal*N*Ac residues was incorporated into a non-proteinaceous molecule within *S.* Typhimurium 82/6915*ΔaroA::pgl,* most probably LPS.

**Figure 3:**
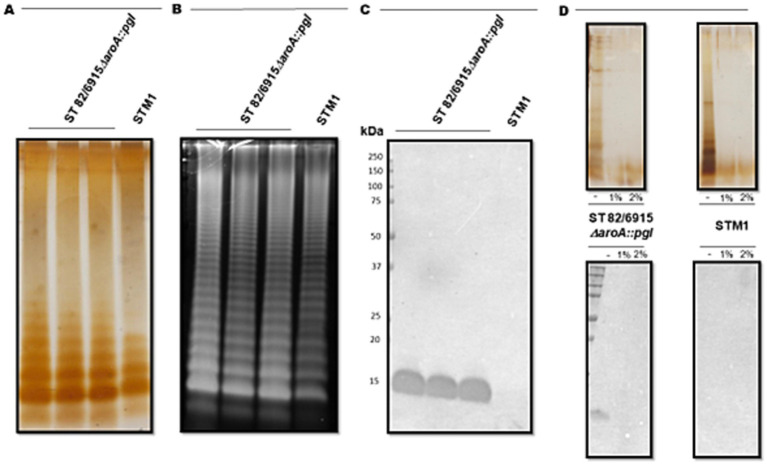
Visualisation and analysis of *Salmonella* Typhimurium 82/6915*ΔaroA::pgl* (three independent cultures) and STM-1 LPS extracts, separated by SDS-PAGE and stained or transferred to a PVDF membrane for lectin blotting. **(A)** Silver-stained LPS extracts. **(B)** Pro-Q Emerald 300 Lipopolysaccharide stained LPS extracts. **(C)** SBA lectin blot of LPS extracts. **(D)** Mild acid hydrolysis (1% or 2% AcOH) of silver-stained (top panel) and lectin-blotted (bottom panel) LPS extracts.

To confirm the 15 kDa signal corresponded to the modification of *S.* Typhimurium 82/6915*ΔaroA::pgl* LPS with a *Campylobacter* saccharide containing Gal*N*Ac residues, LPS was purified and analyzed (Figure 3).

*S.* Typhimurium 82/6915*ΔaroA::pgl* and STM-1 LPS were extracted from vaccine strains and visualized by silver staining (Figure 3A). However, silver staining did not indicate whether the *Campylobacter* saccharide had been linked to the *S.* Typhimurium 82/6915*ΔaroA::pgl* endogenous LPS and impacted the polysaccharide structure of the endogenous LPS, specifically the O-antigen region. Pro-Q Emerald 300-stained LPS extracts revealed that the addition of the *Campylobacter* saccharide had no impact on the polysaccharide region of *S.* Typhimurium 82/6915*ΔaroA::pgl* LPS, demonstrated by the presence of intact O-antigen repeating units attached to the core-oligosaccharide (Figure 3B). SBA lectin blotting produced a dominant signal at 15 kDa (Figure 3C) and confirmed the incorporation of a *Campylobacter* saccharide that contained at least one Gal*N*Ac residue as a part of *S.* Typhimurium 82/6915*ΔaroA::pgl* LPS. To better understand which domain of *S.* Typhimurium 82/6915*ΔaroA::pgl* LPS the *Campylobacter* saccharide was incorporated into, mild-acid hydrolysis of LPS was performed (Figure 3D). The 15 kDa SBA-positive band was sensitive to mild acid hydrolysis, which resulted in hydrolysis of the Kdo residue and the subsequent loss of the 15 kDa SBA signal. Loss of the SBA signal indicated that the *Campylobacter* saccharide was incorporated into *S.* Typhimurium 82/6915*ΔaroA::pgl* LPS.

#### 3.1.2 Determination of the carbohydrate structure of the LPS-heptasaccharide glycoconjugate

**Figure 4:**
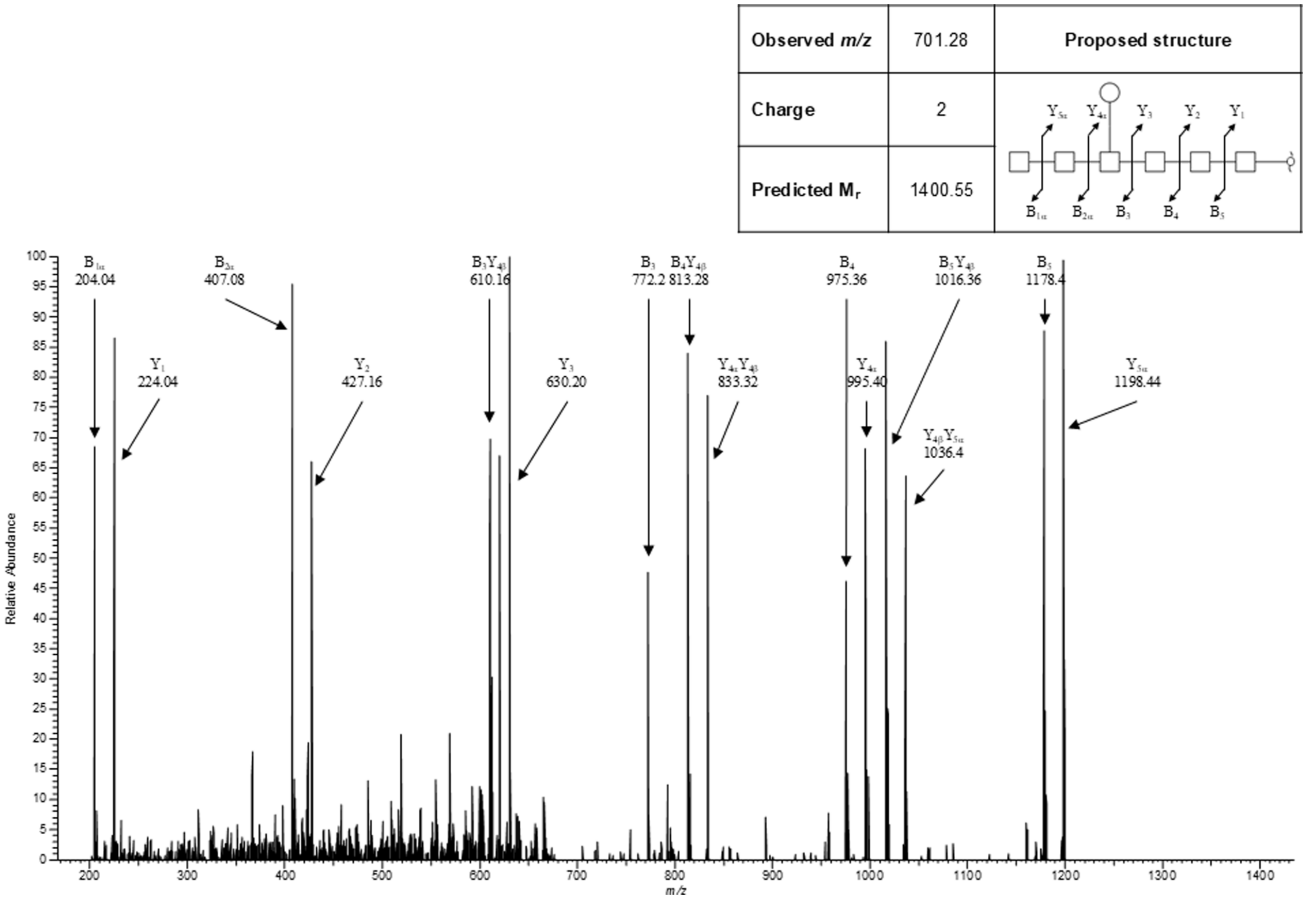
Representative MS2 spectra for the heptasaccharide structure unique to *Salmonella* Typhimurium 82/6915*ΔaroA::pgl*.

To determine the structure of the *S.* Typhimurium 82/6915*ΔaroA::pgl* LPS-*Campylobacter* carbohydrate conjugate, the polysaccharide region of *S.* Typhimurium 82/6915*ΔaroA::pgl* LPS and unmodified STM-1 LPS were cleaved from the lipid-A core, filtered, and analyzed by LC–MS/MS. Analysis using LC–MS/MS detected a heptasaccharide species unique to *S.* Typhimurium 82/6915*ΔaroA::pgl*. Analysis of MS2 fragmentation spectra for this heptasaccharide confirmed that it is composed of a linear chain of six HexNAc residues with a single branching Hex attached to the 4th HexNAc from the reducing terminus (Figure 4). This structure is consistent with the well-characterized *C. jejuni* heptasaccharide. However, one sugar residue differed whereby the reducing end, which typically consists of di*N*AcBac, had been substituted with a HexNAc. Fragment series were annotated in concordance with the nomenclature established by Doman and Costello (56). This heptasaccharide structure was absent in STM-1 LPS (Supplementary Figure 1), which served as a negative control for the *Campylobacter* heptasaccharide modification.

**Figure 5:**
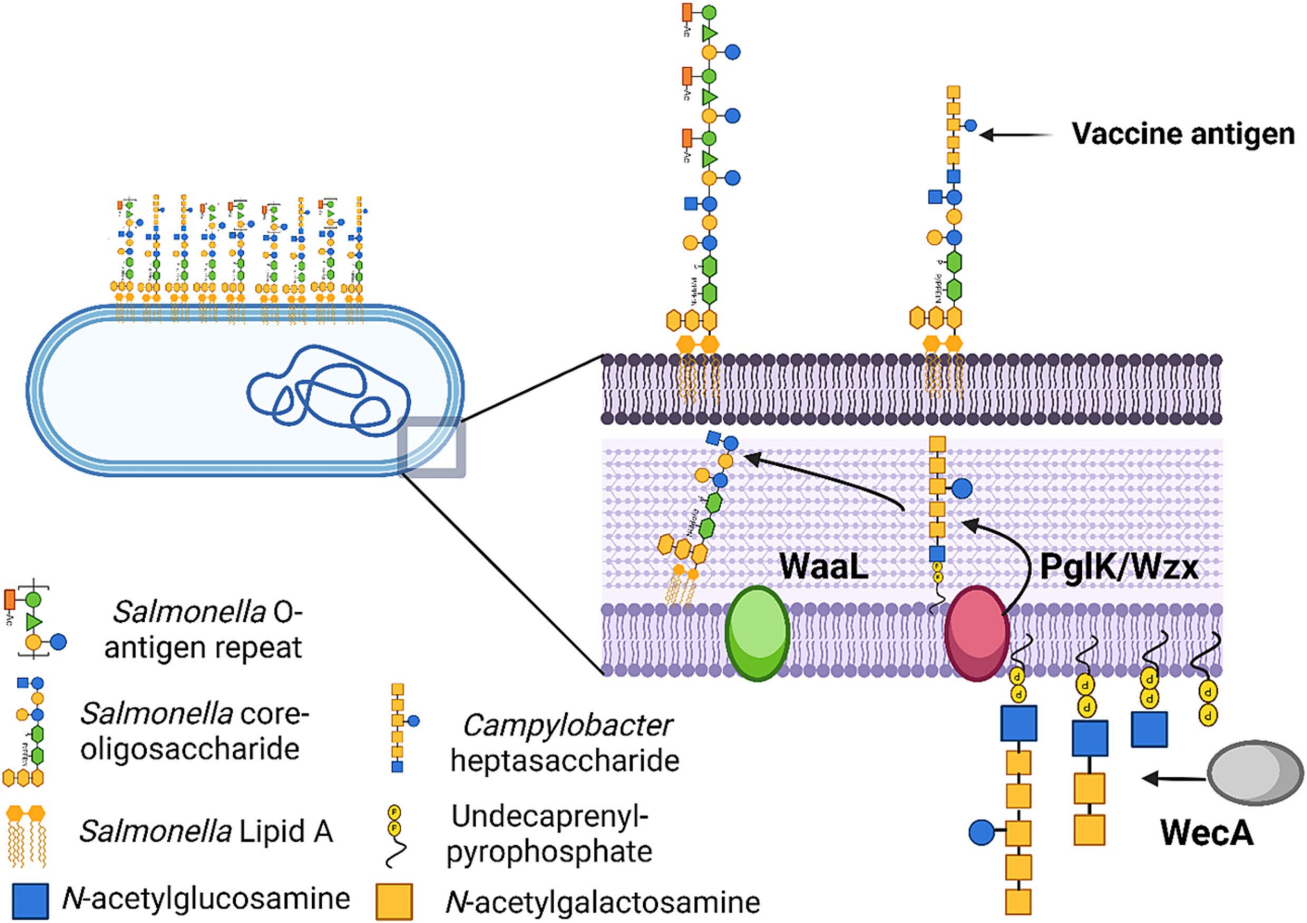
Schematic illustration of *Salmonella* Typhimurium 82/6915*ΔaroA::pgl* vaccine strain and the process by which the *Campylobacter* heptasaccharide is integrated into *Salmonella* LPS. Created in BioRender. McDonald, J. (2024) BioRender.com/r26w279.

These results indicated that the *Campylobacter* heptasaccharide was incorporated into *S.* Typhimurium 82/6915*ΔaroA::pgl* LPS onto the core-oligosaccharide region as depicted in Figure 5.

### 3.2 Surface display of the *Campylobacter* heptasaccharide in *Salmonella* Typhimurium 82/6915*ΔaroA::pgl*

**Figure 6:**
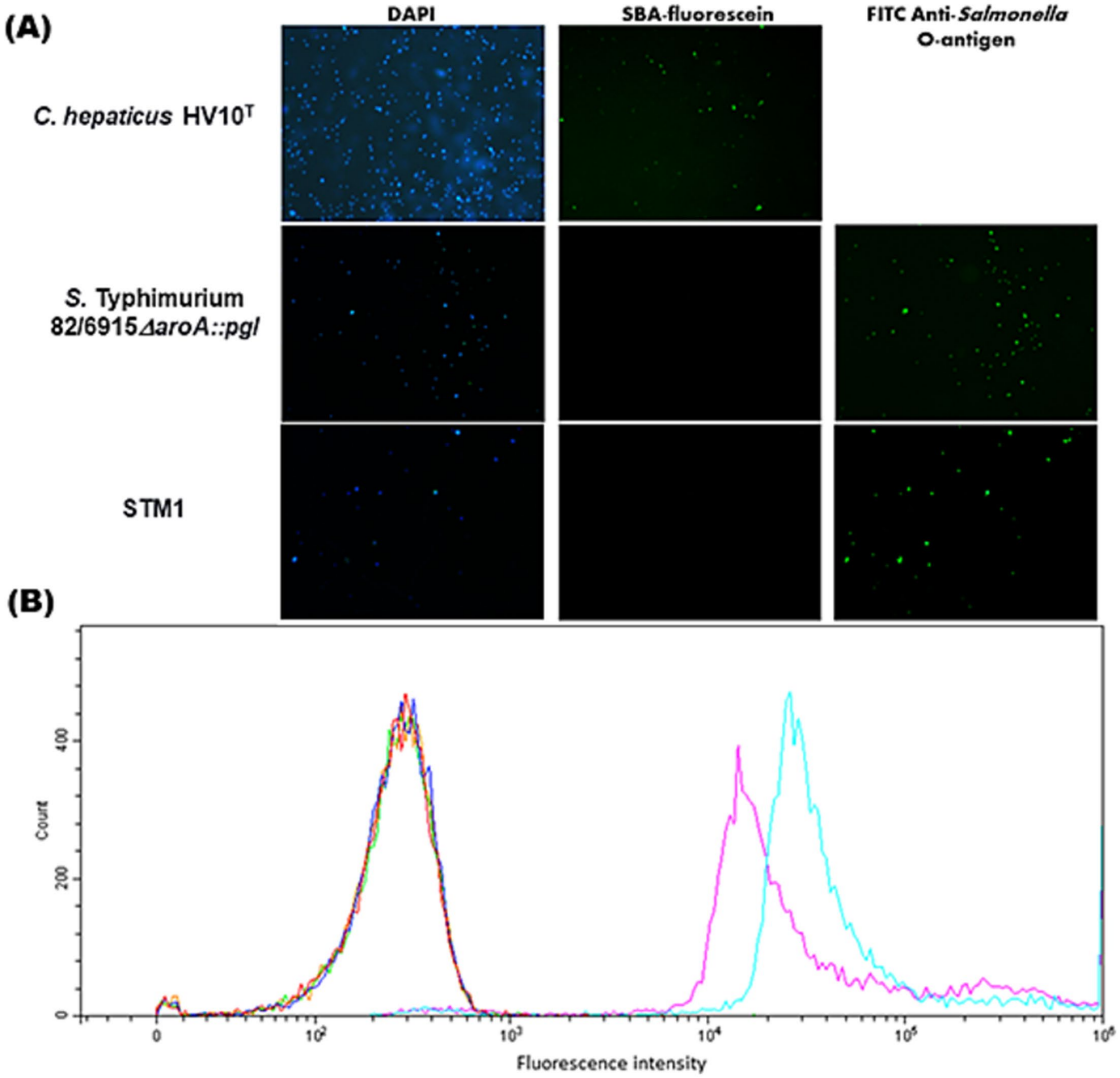
Fluorescence microscopy and flow cytometry to assess surface display of the *Campylobacter* heptasaccharide in *Salmonella* Typhimurium 82/6915*ΔaroA::pgl*. **(A)** Fluorescence microscopy of OD_600_ = 0.3 overnight fixed cultures of *Salmonella* Typhimurium 82/6915*ΔaroA::pgl* and *Salmonella* Typhimurium 82/6915 or OD_600_ = 0.3 48 h cultures of *C. hepaticus* HV10^T^ (Panel A) were blocked and stained with Gal*N*Ac-recognising lectin SBA conjugated to fluorescein or FITC anti-*Salmonella* antibody. Samples nuclei were counterstained with 4′,6-diamidino-2-phenylindole (DAPI). **(B)** Flow cytometric analysis was acquired and analyzed on a Beckman Cytoflex using CytExpert 2.5 software. The equivalent of OD_600_ = 0.3 overnight fixed cultures of *Salmonella* Typhimurium 82/6915*ΔaroA::pgl* and *Salmonella* Typhimurium 82/6915 were blocked and then left unstained (orange and green, respectively) or probed with either SBA-fluorescein (dark blue and red, respectively) or FITC anti-*Salmonella* antibody (light blue and purple, respectively). Stained samples were diluted 1/1000 in PBS, and data were presented as histograms. A total of 10,000 events were analyzed for each sample.

Although the *Campylobacter* heptasaccharide was confirmed to be incorporated into *S.* Typhimurium 82/6915*ΔaroA::pgl* LPS, it was speculated that the display of the antigen at the cell surface might be limited due to the presence of the endogenous polysaccharide O-antigen domain, which is considerably larger than the *Campylobacter* heptasaccharide. Therefore, flow cytometric and fluorescence microscopic analysis of cells stained with fluorescently labeled SBA and FITC anti-*Salmonella* O-antigen antibody were used to investigate the surface display of the *Campylobacter* heptasaccharide. Fluorescence microscopy of *S.* Typhimurium 82/6915*ΔaroA::pgl* with fluorescein labeled SBA produced no fluorescent signal and indicated surface display of the heptasaccharide was hidden (Figure 6A). In comparison, staining of *C. hepaticus* HV10^T^ produced a strong fluorescence signal, which likely corresponded to SBA-fluorescein binding to the heptasaccharide on surface-exposed glycoproteins. The absence of surface exposure of the *Campylobacter* heptasaccharide on *S.* Typhimurium 82/6915*ΔaroA::pgl* cells was supported by staining with fluorescently labeled anti-*Salmonella* O-antigen antibody, which produced strong fluorescent signals.

Flow cytometric of *S.* Typhimurium 82/6915*ΔaroA::pgl* and *C. hepaticus* HV10^T^ cells supported these findings (Figure 6B), suggesting that the *Campylobacter* heptasaccharide is obscured by the *S.* Typhimurium 82/6915*ΔaroA::pgl* O-antigen domain of its endogenous LPS.

### 3.3 Assessment of the immunological characteristics of the *Salmonella* Typhimurium 82/6915*ΔaroA::pgl* vaccine candidate in layer hens

All birds were negative for *C. hepaticus, C. jejuni,* and *S.* Typhimurium prior to transfer to the facility, and the negative controls (Group 1) remained free of *C. hepaticus, C. jejuni,* and *S.* Typhimurium on cloacal swabs collected at weekly intervals throughout the study.

#### 3.3.1 Vaccine persistence and re-isolation

An animal trial was conducted to investigate the persistence/attenuation status and to identify and quantify the humoral immune responses induced following intramuscular injection of *S.* Typhimurium 82/6915*ΔaroA::pgl.* Groups (Table 1) of 15-layer hens were vaccinated via intramuscular injection at 7 (day 0) and 11 weeks (day 29) of age. To understand the capacity of vaccine strains to colonize specific sites of the host and the persistence at these sites, tissue and caecal content samples were taken, and vaccine strains were identified by culturing or DNA extraction followed by PCR analysis. At days two and three post-vaccination, *S.* Typhimurium 82/6915*ΔaroA::pgl* was re-isolated from the injection site (breast muscle) of all five birds by culturing and was detected by PCR in group 3 vaccinated birds (Table 3). On day two post-vaccination, STM-1 was detected in the breast muscle of four out of five birds in group two, but by day three, this had reduced to three out of five birds re-isolated by culturing; however, it was detected in all birds by PCR. On day two post-vaccination, neither vaccine strain was detected in necropsied birds’ caecal content, liver, or spleen (Table 4). On day three post-vaccination, STM-1 was detected by PCR in the caecal content of two birds from the STM-1 vaccinated group, whereas *S.* Typhimurium 82/6915*ΔaroA::pgl* was not detected in the caecal content of any birds (Table 4). Neither vaccine strain was detected in the liver or spleen of necropsied birds on days two and three post-vaccination (Table 5). At day 42, neither vaccine strain nor any tissue samples or caecal content were isolated. In addition, vaccines were not detected by PCR.

**Table 3:** Breast muscle culture and PCR detection of vaccines 2 and 3 days post-vaccination.

	Controls			Vaccinated groups					
	No template control	Vaxsafe^®^ ST	ST 82/6915*ΔaroA::pgl*	Group 1: Unvaccinated		Group 2: Vaxsafe^®^ ST		Group 3: ST 82/6915*ΔaroA::pgl*	
Study day post-vaccination				D2	D3	D2	D3	D2	D3
Culturing	N/A	+	+	0/5	0/5	4/5	3/5	5/5	5/5
panST qPCR	−	+	N/A	0/5	0/5	5/5	5/5	5/5	5/5
Vaxsafe^®^ ST qPCR	−	+	−	N/A	N/A	5/5	5/5	0/5	0/5
ST*aroA::pgl* PCR	−	−	+	0/5	0/5	0/5	0/5	5/5	5/5

**Table 4:** Caecal content culture and PCR detection of vaccines 2 and 3 days post-vaccination.

	Controls			Vaccinated groups					
	No template control	Vaxsafe^®^ ST	ST 82/6915*ΔaroA::pgl*	Group 1: Unvaccinated		Group 2: Vaxsafe^®^ ST		Group 3: ST 82/6915*ΔaroA::pgl*	
Study day post-vaccination				D2	D3	D2	D3	D2	D3
Culturing	N/A	+	+	0/5	0/5	0/5	0/5	1/5	0/5
panST qPCR	−	+	N/A	0/5	0/5	0/5	2/5	1/5	0/5
Vaxsafe^®^ ST qPCR	−	+	−	N/A	N/A	N/A	2/5	0/5	N/A
ST*aroA::pgl* PCR	−	−	+	0/5	0/5	0/5	0/5	0/5	0/5

**Table 5:** Liver and spleen PCR detection of vaccines 2 and 3 days post-vaccination.

	Controls			Vaccinated groups		
	No Template Control	Vaxsafe^®^ ST	ST 82/6915*ΔaroA::pgl*	Group 1: Unvaccinated	Group 2: Vaxsafe^®^ ST	Group 3: ST 82/6915*ΔaroA::pgl*
panST qPCR	-	+	N/A	0/5	0/5	0/5

#### 3.3.2 *Vaccination with Salmonella* Typhimurium 82/6915*ΔaroA::pgl* produces IgY responses specific to the *Campylobacter* heptasaccharide region of the LPS-heptasaccharide glycoconjugate

**Figure 7:**
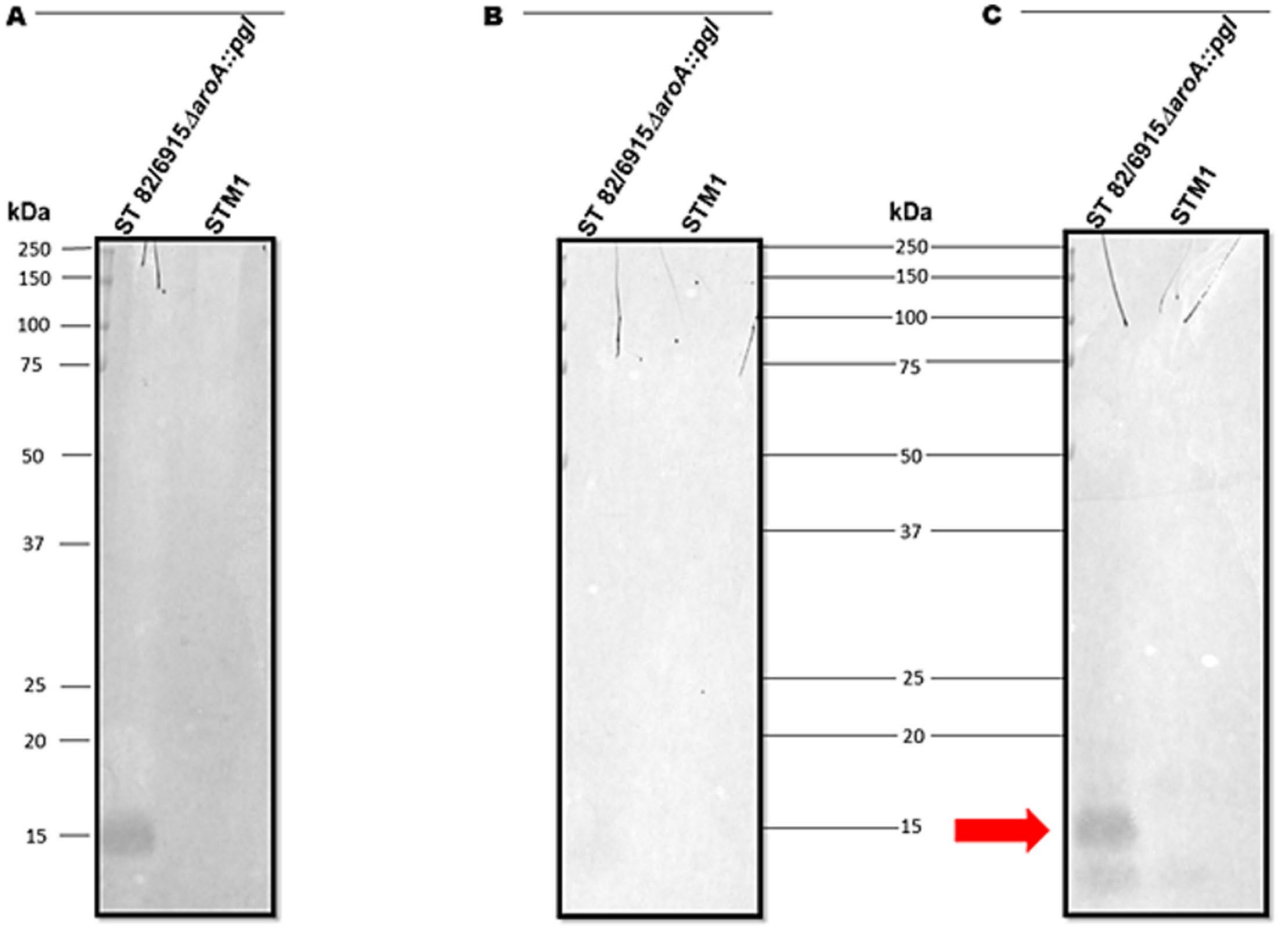
Immunoblot analysis of IgY antibodies in pooled chicken sera at day 42 post-vaccination. **(A)** The SBA lectin blot of *Salmonella* Typhimurium 82/6915*ΔaroA::pgl* and STM-1 LPS extracts confirmed the glycosylation status of the vaccine. **(B)** Immunoblot of *Salmonella* Typhimurium 82/6915*ΔaroA::pgl* and STM-1 LPS extracts against day 42 pooled chicken sera from unvaccinated birds. **(C)** Immunoblot of *Salmonella* Typhimurium 82/6915*ΔaroA::pgl* and STM-1 purified LPS against day 42 pooled chicken sera from the *Salmonella* Typhimurium 82/6915*ΔaroA::pgl* vaccinated birds. The immunodominant band, which corresponded to *Salmonella* Typhimurium 82/6915*ΔaroA::pgl* LPS region modified with the *Campylobacter* heptasaccharide, is highlighted (red arrow).

To determine if vaccination with *S.* Typhimurium 82/6915*ΔaroA::pgl* produced an antigen-specific IgY immune response to the *Campylobacter* heptasaccharide, pooled chicken sera taken at day 42 from birds vaccinated twice with *S.* Typhimurium 82/6915*ΔaroA::pgl* were analyzed by immunoblotting against purified *S.* Typhimurium 82/6915*ΔaroA::pgl* LPS altered at the core-oligosaccharide with the *Campylobacter* heptasaccharide. IgY antibodies in the pooled sera bound to the LPS-*Campylobacter* heptasaccharide glycoconjugate produced an immunodominant signal at 15 kDa (Figure 7). This signal was absent in pooled sera from day 42 unvaccinated birds, which indicated the immunodominant band corresponded to a *Campylobacter* heptasaccharide-specific IgY immune response.

**Figure 8:**
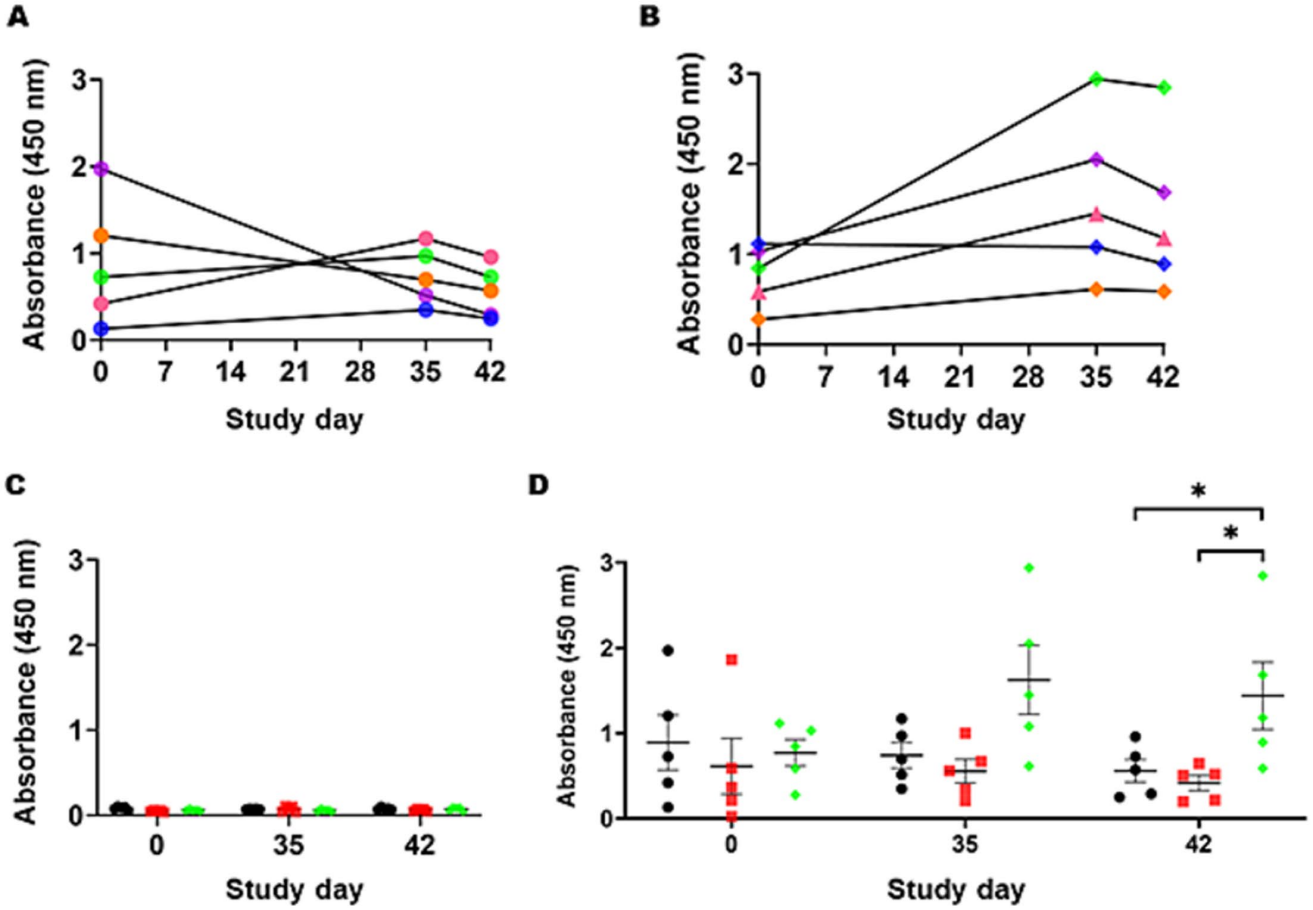
*Campylobacter* heptasaccharide-specific IgY antibody responses to intramuscular vaccination with *Salmonella* Typhimurium 82/6915*ΔaroA::pgl*. **(A)** Individual bird IgY titers against the heptasaccharide in mock-vaccinated birds at day 0, 35, and 42 post-vaccination are illustrated with each bird represented by a unique color: B36 in orange, B37 in blue, B38 in pink, B39 in green, B40 in purple IgY titres against the heptasaccharide in mock vaccinated birds at day 0, 35, and 42 post-vaccination. **(B)** IgY titers for individual birds in the *Salmonella* Typhimurium 82/6915*ΔaroA::pgl* vaccinated group: B81 in orange, B82 in blue, B83 in pink, B84 in green, B85 in purple. **(C)** Comparison of IgY titers in the sera of mock-vaccinated birds (black circles), STM-1 vaccinated birds (red squares), and *Salmonella* Typhimurium 82/6915*ΔaroA::pgl* vaccinated birds (green diamonds) against unglycosylated CjaA_ChuA (9). Pre-adsorption of sera with CjaA_ChuA (9) significantly reduced any background responses to CjaA_ChuA (9), demonstrating the specificity of the antibody responses. **(D)** Corrected IgY titers, calculated as the average absorbance values of glycosylated CjaA_ChuA (9) minus the average absorbance values of unglycosylated CjaA_ChuA (9) from the same sera samples in **(C)**. Data points represent IgY antibody responses measured at an optical density (OD) of 450 nm for each chicken, and bars indicate the standard error of the mean. An asterisk (*) indicates statistically significant differences with with *p*-values of <0.05.

To quantify whether the IgY responses specific to the *Campylobacter* heptasaccharide were significantly different from those of mock and STM-1 vaccinated chickens, a serological ELISA was performed using a purified *Campylobacter* glycoprotein, CjaA_ChuA (9), and its unglycosylated equivalent as capture antigens (Supplementary Figure 2) to compensate for any cross-reactivity directed against the protein. *Campylobacter* heptasaccharide-specific immune responses were quantified by ELISA in sera taken on day zero prior to vaccination and days 35 and 42 post-secondary vaccination. Primary vaccination produced no significant increases in IgY responses to the heptasaccharide. However, significantly elevated *Campylobacter* heptasaccharide-specific IgY antibodies were detected in *S.* Typhimurium 82/6915*ΔaroA::pgl-*vaccinated birds (Figure 8D) post-secondary vaccination at day 42 compared to mock-vaccinated and STM-1-vaccinated chickens (Figure 8D). Humoral immune responses were confirmed to be specific to the heptasaccharide, as low binding was detected in sera probed against the unglycosylated protein in all groups (Figure 8C). Prior to the first vaccination, elevated levels of IgY (absorbance greater than one) were detected in two birds (36 and 40) from the mock-vaccinated control group (Figure 8A) and suggested some of these birds were pre-exposed to *Campylobacter*, which is supported by the decreased levels of IgY titres at days 35 and 42. However, no significant differences were observed among the mock, STM-1, and *S.* Typhimurium 82/6915*ΔaroA::pgl* groups before vaccination. Longitudinal analysis of humoral responses in *S.* Typhimurium 82/6915*ΔaroA::pgl-*vaccinated birds (Figure 8B) demonstrated IgY-specific responses to the heptasaccharide that peaked at day 35, a week post-secondary vaccination.

While a statistically significant difference in anti-*Campylobacter* heptasaccharide IgY antibodies was observed between *S.* Typhimurium 82/6915Δ*aroA::pgl* and both the STM-1 and mock-vaccinated groups, inter-animal variability was observed where some *S.* Typhimurium 82/6915Δ*aroA::pgl* vaccinated birds responded weakly to vaccination (e.g., B81) compared to others (B84 and B85).

#### 3.3.3 Modification of *Salmonella* Typhimurium 82/6915Δ*aroA::pgl* endogenous LPS core-oligosaccharide with the *Campylobacter* heptasaccharide had no significant impact on vaccine immunogenicity toward *Salmonella* Typhimurium

**Figure 9:**
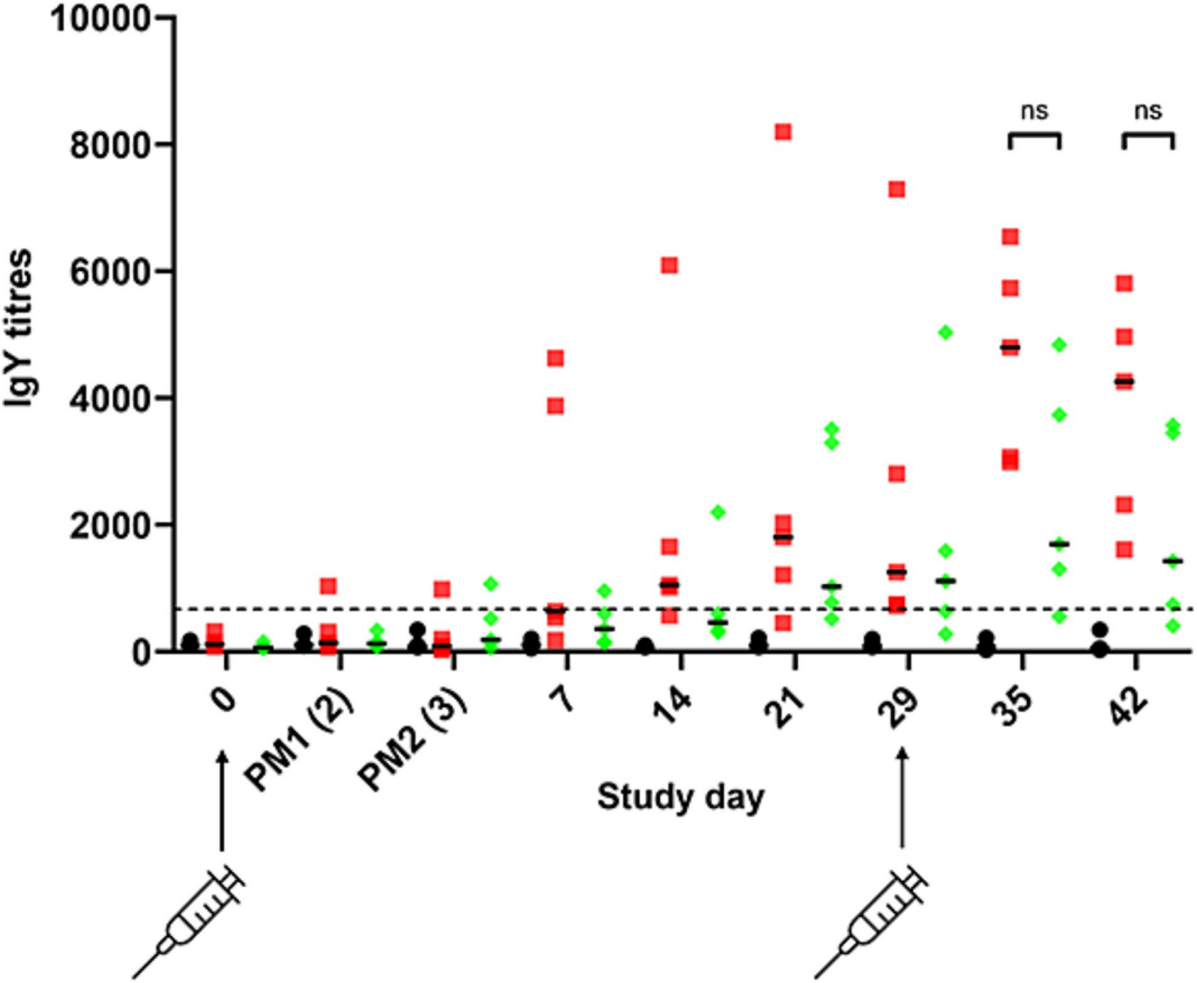
*Salmonella* Typhimurium-specific IgY antibody responses to intramuscular vaccination with STM-1 (red squares)*, Salmonella* Typhimurium 82/6915*ΔaroA::pgl* (green diamonds), or mock vaccinated (black circles). ELISA results were obtained using a 1:100 dilution of chicken sera from bleeds, which were taken weekly across the time course of the trial. Each point represents the antibody response (IgY titre) measured at 450 nm and calculated according to BioChek instructions for each bird. The dotted line represents the cutoff value (654) for an antibody response specific to *Salmonella* Typhimurium provided by BioChek. Any titres below 654 are considered non-specific. Data are presented as individual values and group median values with 95% confidence intervals. Statistical differences between groups are indicated: ns, not statistically significant (*p*-value >0.05).

To determine if modification of *S.* Typhimurium 82/6915Δ*aroA::pgl* endogenous LPS core oligosaccharide with the addition of the *Campylobacter* heptasaccharide impacted immune responses against *S.* Typhimurium, IgY responses specific to *S.* Typhimurium were quantified using ELISA (Figure 9). The primary IgY antibody responses to *S.* Typhimurium were weak after the first vaccination and gradually increased in *S.* Typhimurium 82/6915Δ*aroA::pgl,* and STM-1 vaccinated birds at days seven to 29 relative to mock vaccinated birds. Two weeks (day 14) post-primary vaccination, IgY titres in STM-1 vaccinated birds were significantly higher than in the mock-vaccinated group. Post-secondary vaccination, individual bird antibody titres increased in both *S.* Typhimurium 82/6915Δ*aroA::pgl* and STM-1 vaccinated birds. No detectable anti*-S.* Typhimurium-specific IgY titres were present in mock vaccinated birds. All STM-1 vaccinated birds produced IgY antibody titres above the cutoff of 654, with any values below this deemed non-responsive. One bird from the *S.* Typhimurium 82/6915Δ*aroA::pgl* vaccinated groups had antibody titres below the cutoff value, indicating no production of anti-*S.* Typhimurium antibodies. However, antibody titres in chicken sera against *S.* Typhimurium showed no significant differences between the STM-1 and *S.* Typhimurium 82/6915Δ*aroA::pgl* groups on days 14, 29, 35, and 42 (Figure 9).

## 4 Discussion

The global expansion of the cage-free layer industry is accompanied by an increase in the prevalence of bacterial pathogens such as *C. hepaticus,* which poses a significant risk to cage-free egg production. Poultry vaccination effectively controls bacterial pathogens in layer chickens and reduces the need for antibiotics commonly used in food production systems. However, there are no reports of effective vaccines against *C. hepaticus* in the scientific literature. The pathobiology of *C. hepaticus* is unique among campylobacters, and it is a dedicated poultry pathogen. It colonizes the GI tract and the gallbladder (57). The anatomical connection between the chicken gallbladder and liver is suspected to contribute to SLD pathogenesis (58). Following infection with *C. hepaticus,* clinical signs of disease typically appear within 5–7 days (59). Hence, the induction of an immune response via an appropriate vaccination strategy to prevent systemic infection presents an attractive approach to controlling *C. hepaticus* in the poultry industry due to some key considerations.

1. The trafficking of *C. hepaticus* from the gut to the gallbladder could make it more susceptible to the immune system of layer hens than *C. jejuni,* which commonly resides commensally in the gut lumen.
2. A secretory IgY antibacterial immune response could play a role in the reduction of disease incidence and severity through the prevention of *C. hepaticus* circulation in the bile and colonisation of the gallbladder, a common site of *C. hepaticus* infection.
3. Egg-laying hens have a lifespan of about one year, but SLD outbreaks typically occur between 26 and 32 weeks of age (60). This schedule allows vaccination at an older age, giving the immune system sufficient time to develop before birds face a higher risk of disease.

*C. hepaticus* possesses an *N-*linked general glycosylation system that post-translationally modifies multiple proteins with a heptasaccharide glycan, which is believed to play a crucial role in SLD pathogenesis (25, 46). Targeting this conserved heptasaccharide as an antigen is appealing, as it has demonstrated efficacy in reducing the carriage of closely related species *C. jejuni* in broiler chickens (43, 61). Using a live recombinant attenuated *S.* Typhimurium to express *C. hepaticus* antigens, such as the *Campylobacter* heptasaccharide, could provide both humoral and cellular immunity against the pathogen to prevent disease. Additionally, this approach can be bi-valent, simultaneously controlling *Salmonella* infections in poultry.

Based on the above considerations, this study used *S.* Typhimurium as a vector to deliver the *Campylobacter* heptasaccharide to commercial-layer chickens. The experimental vaccine adopted a new approach using an attenuated *S.* Typhimurium strain with an intact O-antigen region. Previous live bacterial vaccine candidates against *C. jejuni* have used O-antigen mutant background strains of the host bacteria to optimize the display of the *Campylobacter* heptasaccharide (43). While this may be necessary for vaccination against *C. jejuni*, completely replacing the natural O-antigen in *S.* Typhimurium may negatively impact the vaccine’s efficacy toward *Salmonella* (62). The vaccination approach against *C. hepaticus* differs from that of *C. jejuni,* as the goal may not be for *Salmonella* to replicate in the gut of the chicken host (site of infection of *C. jejuni*), where it provides prolonged exposure to the vaccine antigen. Instead, it may be more appropriate to induce a systemic immune response via intramuscular injection, where cells are phagocytosed, eventually lysed, and processed naturally by the host, exposing the *Campylobacter* heptasaccharide to the host’s immune system.

Expression of the *Campylobacter* heptasaccharide and attenuation of the *S.* Typhimurium live vector was achieved by chromosomal integration of the *pgl* locus into *S.* Typhimurium 82/6915 by replacement of the *aroA* gene that is central to metabolism. Expression of the *pgl* locus in *S.* Typhimurium 82/6915 resulted in the incorporation of a *Campylobacter* heptasaccharide onto the core-oligosaccharide region of *S.* Typhimurium 82/6915*ΔaroA::pgl* LPS due to the interplay of *S.* Typhimurium endogenous O-antigen and the *Campylobacter N-*glycan biosynthesis pathways (38–40). However, the structure of the heptasaccharide differed from typical *Campylobacter N-*glycan*-*modified glycoproteins, whereby the reducing end sugar, di*N*AcBac, had been substituted for a HexNAc. This substitution likely corresponded to Glc*N*Ac (where Glc*N*Ac is *N*-acetylglucosamine), which has previously been reported in a study that conjugated the same structure to the core-oligosaccharide region of *E. coli* LPS (39, 43). This is likely due to the activity of *S.* Typhimurium endogenous WecA transferase. WecA catalyses the transfer of the Glc*N*Ac-1-phosphate moiety from UDP-Glc*N*Ac onto the carrier lipid UndP; thus, it competes with PglC for the lipid carrier (33, 39, 63). The *Campylobacter* PglC cannot use UDP-Glc*N*Ac as a substrate; therefore, the formation of UndP-Glc*N*Ac serves as a substrate for the *Campylobacter* glycosyltransferase, PglA, which induces the specific *Campylobacter* heptasaccharide biosynthesis by transfer of the first Gal*N*Ac residue. The replacement of di*N*AcBac may have negatively impacted the immune response against the *Campylobacter N*-glycan, but studies have shown that the immune response against the *N*-glycan is directed against the Gal*N*Ac residues (64, 65) at the terminal end of the heptasaccharide.

Flow cytometry and fluorescence microscopy assessments revealed that the surface display of the heptasaccharide was not detectable in *S.* Typhimurium 82/6915*ΔaroA::pgl*. This lack of detectability could be due to the presence of the endogenous O-antigen region of *S.* Typhimurium LPS, which has a molecular weight greater than 1 million Daltons (35, 66), considerably larger than the 1,400 Dalton *Campylobacter* heptasaccharide (67). Similar observations were previously reported in a live *E. coli* vector expressing the *pgl* locus (35). This finding is further supported by a study using an O-antigen-deficient bacterial strain, which demonstrated sufficient surface display of the heptasaccharide when conjugated to the bacterial vector’s lipid A core (43). It could be questioned why, in this case, an O-antigen mutant background was not used to improve the display of the heptasaccharide. However, this may be inappropriate for the current application to produce a bivalent vaccine, as non-reversible truncation of *S.* Typhimurium O-antigen is not suitable for developing a live vaccine against *S.* Typhimurium due to significantly reduced immune responses to heterologous target antigens associated with O-antigen mutants (62). Despite the poor presentation of the *Campylobacter* heptasaccharide, it was evident that copies of the LPS-*Campylobacter* heptasaccharide glycoconjugate antigen were still exposed to the host immune system, likely in part due to post-lysis of *S.* Typhimurium 82/6915Δ*aroA::pgl in vivo,* as an immune response to the antigen was detected.

Vaccination of commercial free-range layers revealed that replacement of the *aroA* gene by the introduction of the *pgl* locus into *S.* Typhimurium 82/6915 sufficiently attenuated the bacteria, and expression of the *Campylobacter* heptasaccharide in *S.* Typhimurium 82/6915*ΔaroA::pgl* had not impacted bacterial fitness compared to STM-1, as tissue colonisation patterns and persistence were not noticeably different from that of STM-1-vaccinated birds. STM-1 was detected in the caecal content of two out of five birds on day three post-vaccination. However, STM-1 could not be re-isolated from the samples by culturing. Therefore, whether detection was due to cross-contamination or the live vaccine strain passing to the caeca is inconclusive. However, results collectively suggest that when administered via intramuscular injection in the breast muscle, both vaccine strains could not efficiently systemically colonize the birds or pass to the gastrointestinal tract where *S.* Typhimurium typically colonizes and reproduces (2). Therefore, the candidate vaccine strain was transient, and lack of colonisation suggested the host’s immune system had naturally processed *S.* Typhimurium 82/6915*ΔaroA::pgl* via cell lysis and exposed the heptasaccharide to the host’s immune system. Because *S.* Typhimurium 82/6915*ΔaroA::pgl* exhibited similar bacterial fitness and capacity to colonize, disseminate, and persist to the registered vaccine strain STM-1, it is concluded that *S.* Typhimurium 82/6915*ΔaroA::pgl* was sufficiently attenuated to be used safely as a vaccine.

Vaccination with *S.* Typhimurium 82/6915Δ*aroA::pgl* significantly increased IgY antibody responses specific to the *Campylobacter* heptasaccharide. To ensure IgY antibody responses were specific to the heptasaccharide, a highly purified *N-*glycan was used as the capture antigen for ELISA. In addition, sera were preabsorbed with *S.* Typhimurium whole cell lysate and the unglycosylated equivalent of the capture antigen to reduce any background IgY immune responses to *S.* Typhimurium and the carrier protein. Nevertheless, ELISA data revealed high background antibody responses specific to the *Campylobacter* heptasaccharide in some mock and STM-1 birds prior to vaccination, which was speculated to be due to circulating IgY antibodies against the heptasaccharide in birds likely pre-exposed to *Campylobacter* before commencement of the trial. This was not a surprise, as the birds used were sourced from a commercial layer farm. Inter-animal variability of antibody response to vaccination was observed whereby two birds from *S.* Typhimurium 82/6915Δ*aroA::pgl* responded well to vaccination compared to the other birds. Variability may be linked to the method of administration, and it was speculated that co-administration with the EDS-ED adjuvant may have affected vaccine uptake in some birds. The method of vaccine administration was chosen to follow industry standards of STM-1 administration. However, the vaccine may have been more effectively processed by the host’s immune system if administered alone instead of in conjunction with EDS-ND, and future studies should seek to understand this.

As it has now been shown that vaccination with *S.* Typhimurium 82/6915Δ*aroA::pgl* produced IgY heptasaccharide-specific responses, the next step toward SLD vaccine development will be to perform *C. hepaticus* challenge trials in layers to determine if the immune responses can reduce *C. hepaticus* carriage in the gastrointestinal tract or bile and/or reduce the pathogenic consequences of infection. Previous studies have been unable to detect a clear association between antigen-specific IgY and protection against *C. jejuni* (61, 68). However, it is suspected that antigen-specific IgY levels may play a more important role in protection against *C. hepaticus* compared to *C. jejuni* due to its ability to systemically infect the gallbladder and induce disease in the liver, whereas *C. jejuni* commonly resides in the gut of poultry without causing overt disease. Varying levels of anti-*Campylobacter* heptasaccharide IgY-specific responses have been reported between groups. The use of several different subunit glycoconjugate vaccine regimes against *C. jejuni* elicited strong protein antigen-specific IgY responses but failed to detect any significant antibody responses against the heptasaccharide (68–70), whereas a live surface-based glycoconjugate, where the heptasaccharide was fused to the lipid-A core of a bacterial vector, induced significant antibody titres specific to the heptasaccharide (43). A comparison of these studies is challenging due to the inconsistencies in vaccination schedules and delivery, chicken lines used, levels of glycosylation achieved, and variability in the assays used to assess vaccine efficacy. However, previous findings and those presented in this study collectively suggest that conjugation of the heptasaccharide to the LPS of a bacterial vector may be more effective in inducing heptasaccharide-specific antibody responses than when the heptasaccharide is conjugated to a carrier protein and delivered as a subunit vaccine. This emphasises the need to better understand the role the *Campylobacter* heptasaccharide plays in the induction of immune responses.

A comparison of IgY antibody responses directed against *S.* Typhimurium in the sera of STM-1 and *S.* Typhimurium 82/6915Δ*aroA::pgl* vaccinated birds revealed that modification of *S.* Typhimurium 82/6915Δ*aroA::pgl* LPS with the *Campylobacter* heptasaccharide had no significant impact on IgY antibody responses against *S.* Typhimurium. Despite a lack of statistical significance in antibody responses directed against *S.* Typhimurium in the sera of *S.* Typhimurium 82/6915Δ*aroA::pgl* vaccinated birds and STM-1 vaccinated birds, the individual bird antibody titres were noticeably less in *S.* Typhimurium 82/6915Δ*aroA::pgl* vaccinated birds, suggesting that conjugation of the *Campylobacter* heptasaccharide to the core-oligosaccharide region of *S.* Typhimurium LPS may have impacted the level of humoral responses toward *S.* Typhimurium. Further research is required to achieve consistent antibody responses across birds and to conduct efficacy trials. Notably, intramuscular injection into the pectoralis muscle is more laborious and less economical. However, this route of administration has been shown to produce increased levels of circulating antibodies (71) and follows industry standards when administering *S.* Typhimurium vaccines. Additionally, this route is likely the most suitable for the induction of a potent systemic immune response against *C. hepaticus*.

In summary, the data presented in this study provides evidence that the *Campylobacter* heptasaccharide incorporated as a part of the LPS of a live vectored *S.* Typhimurium strain is a rational approach to produce a bi-valent immune response against the *Campylobacter* heptasaccharide and *S.* Typhimurium. The potential of the current vaccine candidate can be tested in an efficacy trial using the appropriate animal model (59).

## Data Availability

Sequence data for the plasmid used to construct the vaccine strain *S.* Typhimurium 82/6915Δ*aroA::pgl* has been deposited in the GenBank Data Libraries under the accession number PV289569.
