# Supplementary material for: Immunological and pathobiological characteristics of a novel live Salmonella Typhimurium-vectored Campylobacter vaccine candidate for layer chickens
Source: Front Vet Sci. 2025 Mar 21;12:1518231. doi: 10.3389/fvets.2025.1518231 (PMC11969459; doi:10.3389/fvets.2025.1518231)
Supplement: Supplementary file 1 [file Data_Sheet_1.docx]

**Supplementary material**


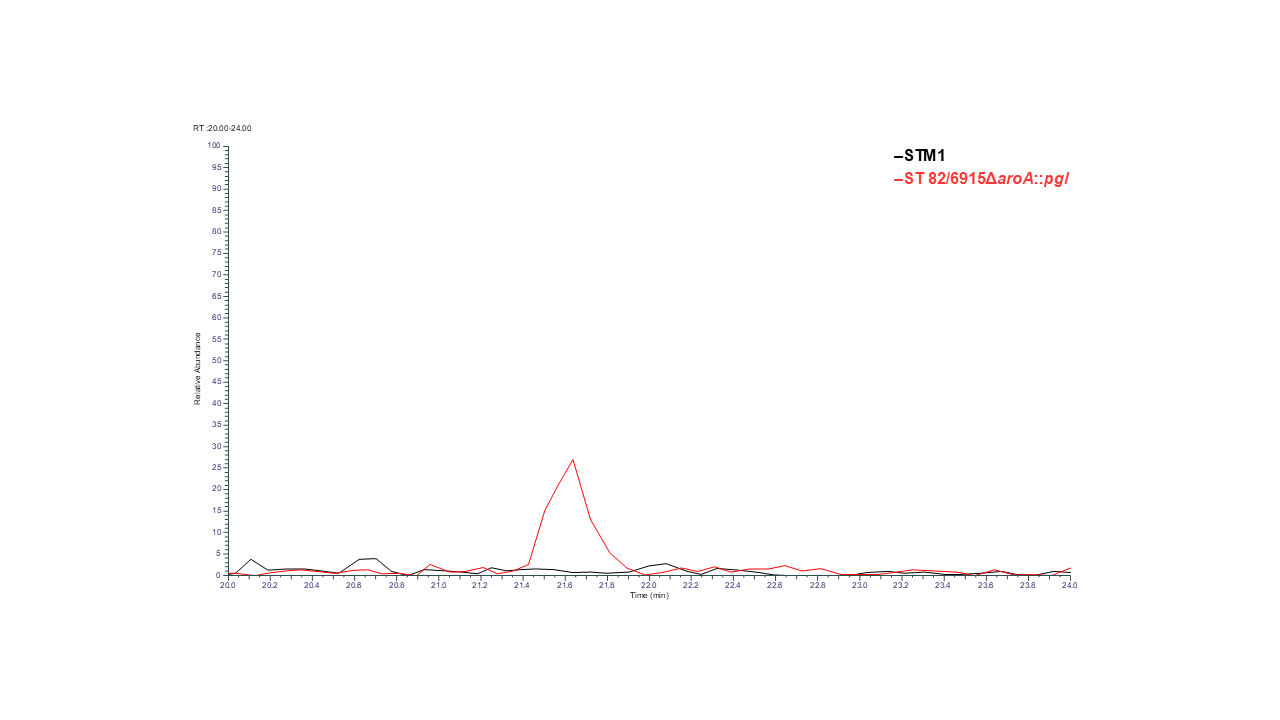


Supplementary Figure 1. Detection of the heptasaccharide structure by extracted ion chromatogram of *m/z* 701.28 in STM1 and *S.* Typhimurium 82/6915 Δ*aroA*::*pgl.*


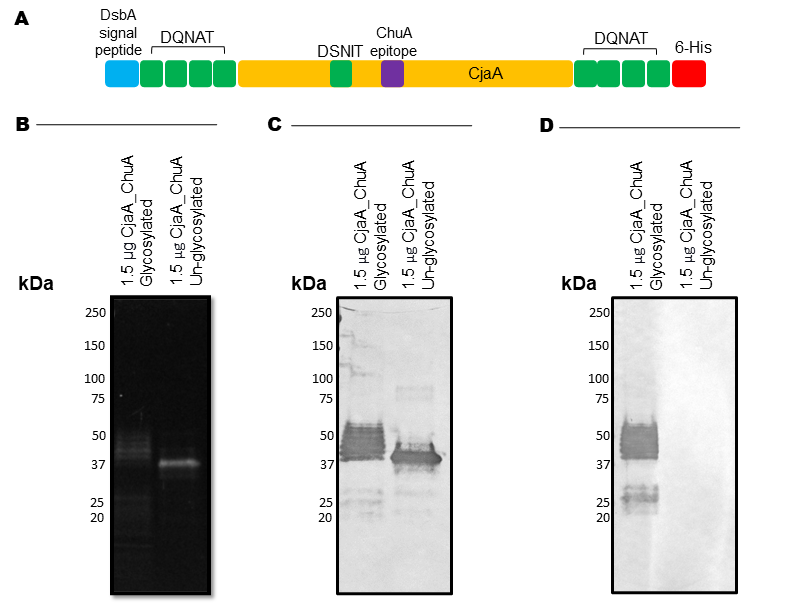


Supplementary Figure 2. Use of the his-tag purified glycoprotein, CjaA_ChuA (9) and its unglycosylated equivalent, CjaA_ChuA as capture antigens to quantify IgY antibody responses to the Campylobacter heptasaccharide (A) Schematic diagram of the CjaA_ChuA (9) recombinant protein: DsbA (blue), periplasmic localisation signal; four glycosylation sequons at the N-terminus (green); CjaA_ChuA integrated fusion protein with 1 naturally occurring glycosylation sequon (green) and the ChuA epitope (purple); four glycosylation sequons at the C-terminus (green); His_6_, hexa-histidine-tag (red). The unglycosylated equivalent, CjA_ChuA lacked the 8 additional DQNAT sequons and was produced in the absence of *pgl* machinery for *N-*glycosylation. (B) Coomassie blue stain of a 4-20% SDS-PAGE of purified proteins. (C) Western blot of purified proteins with mouse anti-his [1:3000]. (D) Lectin blot of the same samples shown in C.
